# Cell wall arabinogalactan is responsible for Fungitell cross reactivity in nocardiosis

**DOI:** 10.1016/j.jbc.2025.110900

**Published:** 2025-11-04

**Authors:** Christophe Mariller, Pascal Letowski, Wei-Ting Chang, Todd L. Lowary, Marc Ulrich, Karine Faure, Séverine Loridant, Boualem Sendid, Frédéric Wallet, Daniel Poulain, Marc Hazzan, Marie Frimat, Yann Guerardel, Marie Titecat

**Affiliations:** 1 Université de Lille, CNRS, UMR 8576 – UGSF - Unité de Glycobiologie Structurale et Fonctionnelle, Lille, France; 2 Institute of Biological Chemistry, Academia Sinica, Taipei, Taiwan; 3 Institute of Biochemical Sciences, National Taiwan University, Taipei, Taiwan; 4 Nephrology Department, University Lille, CHU Lille, Lille, France; 5 Infectious Diseases Department, CHU Lille, Lille, France; 6 CHU Lille, Institute of Microbiology, Lille, France; 7 Laboratoire de Parasitologie-Mycologie, University Lille, CNRS UMR 8576, UGSF, Inserm U1285, CHU Lille, Lille, France; 8 Université de Lille, INSERM, CHU Lille, U1286-INFINITE-Institute for Translational Research in Inflammation, Lille, France; 9 University Lille, Inserm, Institut Pasteur de Lille, U1167 - RID-AGE, Lille, France; 10 Institute for Glyco-core Research (iGCORE), Gifu University, Gifu, Japan

**Keywords:** cross reactivity, nocardiosis, *nocardia nova*, serum (1,3)-β-D-glucan

## Abstract

Nocardiosis is a serious infection in immunosuppressed patients, especially transplant recipients. The slow-growing phenotype of the bacterium and the variety of symptoms complicate diagnosis and delay antimicrobial therapy, resulting in high mortality rates despite effective treatments. A further complication is that some nocardiosis patients test positive in fungal diagnostics that detect (1,3)-β-D-glucan (the Fungitell assay), but the basis for this cross-reactivity remains unknown. We demonstrate that nocardial cell wall arabinogalactan is a cryptic antigen responsible for cross-reactivity in the Fungitell assay and that this antigen is revealed *in vivo* following bacterial cell lysis. We further show that the reactivity results from a β-glucose substitution of the galactan domain, a modification specific to *nocardia*, and identify the optimal antigen as a tetramer of the trisaccharide repeating unit. By providing structural evidence for Fungitell cross-reactivity during nocardiosis, this work paves the way for developing specific diagnostic tools that are currently lacking.

Nocardiosis is a life-threatening, opportunistic infection caused by *Nocardia* spp, a ubiquitous group of Gram-positive environmental bacteria. These organisms are related to mycobacteria through a common structural feature: an arabinogalactan (AG) polysaccharide domain that covalently connects a peptidoglycan layer to mycolic acid (lipid) chains. Among approximately 100 species, *N. nova* complex frequently causes disease in humans (1, 2). Nocardiosis displays various presentations from localized (*i.e.,* skin, lung) to disseminated (*i.e.,* bacteremia, brain abscesses) forms and predominantly occurs in immunocompromised patients, especially those with cellular immune disorders with poor clinical outcome (1, 3). The non-specific clinical features of nocardiosis (4) combined with the slow growing bacterial phenotype can dramatically delay diagnosis. This, in turn, delays the initiation of appropriate antimicrobial therapy – the use of imipenem plus amikacin (5, 6) – an uncommon antimicrobial combination for the management of severe Gram-positive infections.

In addition to nocardiosis, the immunocompromised population is also at risk of developing opportunistic fungal infections with potential serious outcomes. Thus, in case of fever, these patients benefit from fungal antigen monitoring to predict and treat invasive disease without delay (7, 8). Among these antigens, elevations of the β-D-glucan (BDG), a glucose polymer found in the cell wall of many fungal organisms (*i.e., Candida* spp., *Aspergillus* spp., *Pneumocystis* spp.) is indicative of fungal disease (9). The Fungitell assay is based on a biochemical principle that exploits the innate immune response of the horseshoe crab (*Limulus polyphemus*), specifically the activation of a serine protease zymogen known as Factor G, through its ability to recognize β-glucans (10, 11). Factor G is an heterodimer, consisting of two distinct subunits, alpha and beta, which are autocatalytically converted to active factor G in the presence of BDG (10). The structural recognition is dependent not only on the presence of these specific glycosidic linkages but also on the polymeric nature and conformation of the polymer (12). Once bound to the β-glucan, Factor G undergoes a conformational change and autoactivation. This activation converts Factor G into an active serine protease, which then initiates a cascade that culminates in the cleavage of a chromogenic synthetic peptide substrate included in the assay. The intensity of the color change is proportional to the concentration of BDG in the sample, allowing quantitative detection using spectrophotometric methods. Because mammals do not produce BDG, and because such polysaccharides are largely absent from most bacterial cell walls, the presence of β-glucan in human serum serves as a specific biomarker for systemic fungal infection.

Notably, positive BDG assays have been reported in various cases of evolutive nocardiosis (13, 14, 15, 16, 17, 18). However, the mechanisms underlying how a bacterial species that does not produce BDG can give rise to a positive result in diagnostics that detect this antigen have never been reported. Starting from the observation of an apparent BDG elevation in a *N. nova*-infected patient, we identified the AG fraction of the nocardial cell wall as the antigen resulting in BDG test positivity. These results were enhanced by the chemical synthesis of the pattern responsible for the cross reactivity, which both confirmed the structure and provide tools that may help in developing new diagnostic approaches for nocardiosis.

## Results

### The serum from a nocardiosis patient is positive to Fungitell β-D-glucan reagent

**Figure 1:**
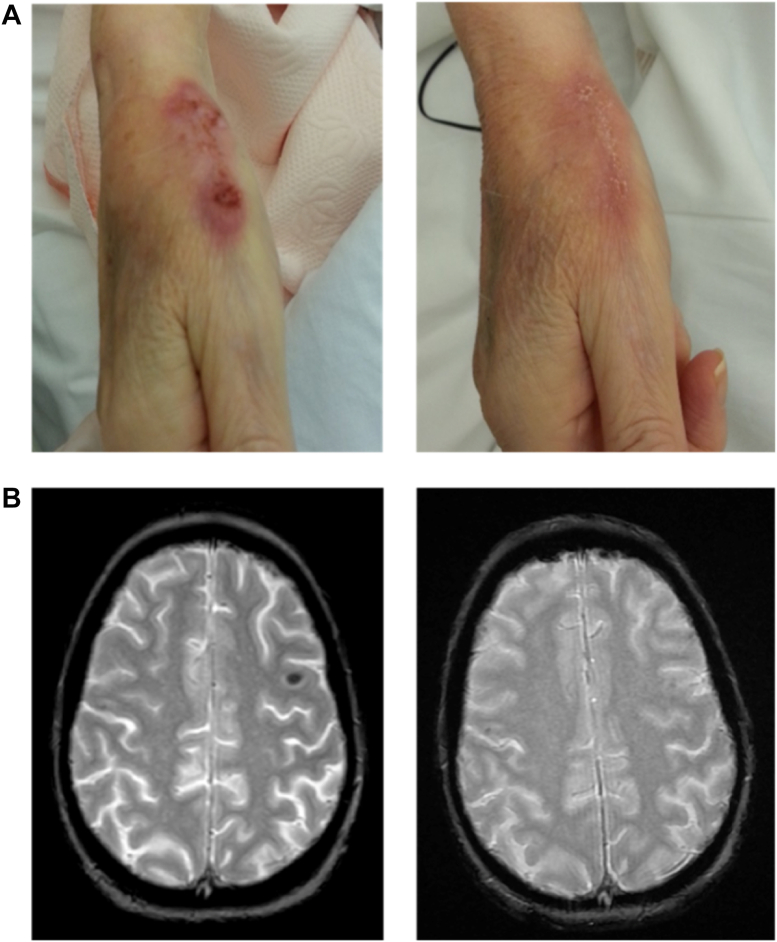
**Patient clinical outcome under optimal antimicrobial therapy.** The evolution of the skin (*A*) and brain abscess (*B*) lesions in the patient were observed over 15 days and 1 month, *left* and *right*, respectively.

A 67-year-old kidney transplant patient was admitted with fever and a month-long ulceration on the right hand associated with an axillary lymph node following gardening (Fig. 1*A*). Immunosuppressive therapy included tacrolimus, mycophenolate mofetil, and steroids. Standard cutaneous and blood cultures yielded negative results, as did PCR assays for *Bartonella* spp. and *Mycobacterium tuberculosis*. A Fungitell serum test exhibited apparent BDG titers of 2000 pg/ml. Skin biopsy analyses showed nonspecific inflammation, without fungal filaments, and 18S PCR assay detected no fungal DNA. Fungitell positive samples tested negative for circulating trehalose (MS-DS), excluding invasive candidiasis (19). First-line therapy with amoxicillin/clavulanate and azithromycin failed to improve symptoms, with apparent BDG levels rising to 2326 pg/ml. Prolonged culture of the surgically removed axillary lymph node showed dry white colonies of branching gram-positive rods identified as *Nocardia nova* (reliability score of 1.95). Brain MRI revealed a cerebral abscess (Fig. 1*B*). Appropriate antimicrobial therapy consisting imipenem (4 weeks) and oral trimethoprim-sulfamethoxazole (12 months), along with adjustments to the immunosuppressive regimen, led to symptom resolution (Fig. 1, *A* and *B*) and a decrease of serum BDG concentrations. Patient features detailed in Table S1.

### The positive Fungitell result is not recovered by direct nocardia cross-reactivity testing

Because the apparent BDG elevation was not associated with any evolutive fungal disease, the *N. nova* clinical strain was directly tested for the possibility of cross reactivity with the Fungitell assay (Fungitell, Cape Cod) according to the Koncan *et al.* procedure (13). Bacterial cells were washed seven times and the inoculum adjusted to 6 × 10^7^ CFU/ml in glucan-free water. Application of the Fungitell test to the suspensions provided negative results (*i.e.,* < 80 pg/ml) even after pellet grinding the pellet content with glass beads on MaGNa Lyser (Roche, Boulogne-Billancourt). A second set of experiments consisting of bacterial growth in glucan-free serum of donor secondarily tested with the Fungitell assay remained negative. These findings invalidated the possibility that the interaction between serum proteins and *N. nova* cell wall elements led to a false positive Fungitell result.

### *Nocardia nova* cell wall extraction is required to identify the positive cell wall pattern

**Figure 2:**
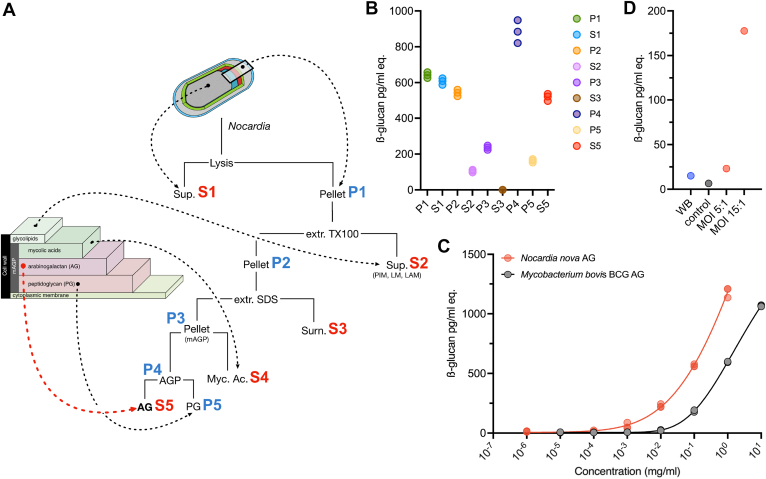
**Subcellular fractionation of *N. nova* and BDG assays.***A,* cell wall components were separated and purified from bacteria through sequential extractions using Triton X100, hot SDS and 2% KOH and centrifugation. *B,* individual fractions (three replicates) were tested using the Fungitell assay and apparent β-(1,3)-D-glucan concentrations are expressed in pg/ml eq. Purified arabinogalactan-peptidoglycan (P4) and arabinogalactan (AG) (S5) show high reactivity to the Fungitell assay. *C,* AG isolated from *N. nova* shows much higher Fungitell reactivity than AG isolated from *M. bovis* BCG (two replicates). *D,* following infection by *N. nova* (MOI 1:5 and 1:15) and processing by macrophage-like THP-1, the culture medium is highly reactive to the Fungitell assay compared with noninfected THP-1 (*control*) and intact, whole bacteria *N. nova* (WB). AG, arabinogalactan.

Because the lack of Fungitell reactivity of intact and ground bacterial pellets apparently contradicted the presence of a cell-surface cross-reactive component, we hypothesized that the component may be unmasked during the infection process. Thus, to identify potential cryptic Fungitell-reactive motifs, *N. nova* cell wall components were sequentially extracted from intact bacteria and individually tested. The *N. nova* cell wall was fractionated following the procedure of Besra and coworkers (20) from an 1196 mg sample of lyophilized bacteria cultivated in Sauton broth (Fig. 2*A*). Fungitell reactivities of each fraction in pg of β-glucan per mL equivalent are reported in Figure 2*B*.

Mechanical lysis of the bacteria in PBS and subsequent centrifugation generated, on the one hand, a supernatant fraction (S1) mainly composed of soluble proteins and a complex mixture of surface components and, on the other hand, a pellet P1. Both S1 and P1 exhibited significant reactivity to the Fungitell assay. At this stage, the S1 fraction was disregarded to avoid possible contamination from surface-associated molecules to focus on bacteria-associated molecules. This was deemed a valid approach as the bacteria themselves were unreactive (above), suggesting a surface-associated molecule was not the cross-reactive species. The reactive P1 fraction was thus further treated with 2% Triton X-100 to extract amphiphilic glycolipids including phospho-inositol mannosides, lipomannan and lipoarabinomannan in the S2 fraction that exhibited low reactivity to the Fungitell assay. In contrast, the resulting pellet fraction (P2) showed very high Fungitell reactivity, comparable to P1, demonstrating that the cross-reactive motif present in P1 was retained in this fraction. Successive extractions of cell-wall associated proteins with hot SDS (P3 and S3) and saponification of mycolic acids using 2% KOH generated an arabinogalactan-peptidoglycan (AGP) fraction (P4) and a lipid fraction that contained mycolic acids (S4). Purified AGP devoid of mycolic acids showed the highest reactivity to Fungitell of all fractions. Finally, dissociation of peptidoglycan (PG) from AGP fraction (P4) led to the isolation of a highly purified AG fraction (S5) that could be structurally defined. S5 retained a high value for apparent BDG (535 pg/ml) albeit slightly lower than P4. In comparison, the PG fraction showed much lower reactivity.

Considering the high reactivity of the AG fraction, we assayed AG that was purified from *Mycobacterium bovis* BCG through the same procedure. At an equivalent concentration, the AG extracted from *M. bovis* BCG demonstrated significantly reduced reactivity, with a concentration shift of over one logarithmic unit compared to the one purified from *N. nova* (Fig. 2*C*). Taken together, these experiments demonstrated that the reactivity of *N. nova* is triggered exclusively after bacterial lysis and that the cell-wall AG is the most likely component responsible for the Fungitell reactivity.

AG is physiologically inaccessible when buried in the bacterial cell wall. It is therefore reasonable to hypothesize that the otherwise cryptic polysaccharide epitopes are exposed after lysis of *N. nova* by immune cells *in vivo*. To test this hypothesis *in vitro*, THP-1 macrophages were infected with labeled *N. nova* at MOIs of 5:1 and 15:1. After 6 hours of infection followed by a 12 hours macrophage incubation, the proportion of phagocytes observed reached 30% with a maximum of two bacteria per cell. At the end of the incubation period, the reactivity of the resulting medium toward β-glucans was evaluated with the Fungitell assay, and compared to medium from control experiments with THP-1 cells alone and *N. nova* alone. As shown in Figure 2*D*, the media collected after infection of THP-1 with *N. nova* showed a much higher reactivity toward β-glucans, in particular at a MOI of 15:1. This result strongly suggests that *N. nova* antigens must be processed by macrophage-induced cell lysis to reveal the cross-reactive antigen, consistent with the lack of reactivity of intact bacteria.

### The AG isolated from *Nocardia nova* is substituted with β-D-glucopyranose residues

To understand the mechanism that underpins the unexpected and specific cross-reactivity of the AG containing fraction isolated from *N. nova* (NnAG) in the Fungitell test, the structure of the reactive fraction (S5) was investigated in detail. AG is the central component of the complex cell wall of Corynebacteriales, including the *Mycobacteriaceae*, *Corynebacteriaceae* and *Norcardiaceae*. Although the structure and biosynthetic pathways of AG have been thoroughly studied in a number of mycobacterial species, information about its composition and fine structure in *nocardia* is scarce. Earlier preliminary studies by Daffe *et al.* suggest that *Nocardia asteroides* and *Nocardia brasiliensis* do synthesize AG with structures that differ significantly from those produced by mycobacteria (21). Monosaccharide composition analysis of fraction S5 isolated from *N. nova* NnAG showed that NnAG was mostly composed of arabinose (Ara) and galactose (Gal), similar to the AG isolated from *M. bovis BCG* (MbAG) as shown in Table 1 (22). Furthermore, it contained a small amount of rhamnose and *N*-acetyl-glucosamine (GlcNAc), strongly suggesting that NnAG is anchored to PG through the Rha*p*-(1,4)-Glc*p*NAc-P disaccharide as in mycobacteria (23). However, unlike mycobacterial AG, NnAG also contained a significant amount of glucose (Glc, 8.5%). Linkage analysis of the monosaccharides established that NnAG contains terminal, 2-, 5-, 3,5- and 2,5-linked Ara in furanose forms (Ara*f*), indicating that the arabinan domain of NnAG shows strong similarities with that of MbAG (Table 1). The Gal residues were observed as 5 galactofuranose (5Gal*f*) and 5,6Gal*f*, demonstrating that the galactan domain of NnAG differs from MbAG, which is composed of a linear chain of alternating 6Gal*f* and 5Gal*f* substituted in the 5 position by the arabinan chain. Finally, all Glc residues were identified as unsubstituted in the pyranose form (Glc*p*) revealing that they substitute AG in terminal positions.

**Table 1:** Monosaccharide composition analysis of arabinogalactan isolated from N. nova (NnAG)

A	
MS	Proportions (%)
Rha	0.8
Ara	64.7
Man	2.9
Glc	8.5
Gal	22.7
GlcNAc	0.3


B	
Linkages	Proportions (%)
t-Ara*f*	5.1
2-Ara*f*	4.2
5-Ara*f*	47
t-Man*p*	0.2
t-Glc*p*	4.3
t-Gal*f*	2.1
3,5-Ara*f*	9.5
2,5-Ara*f*	1.5
5-Gal*f*	13.5
5,6-Gal*f*	12.6

(A) GC- flame ionization detection identification and relative quantification of reduced-peracetylated monosaccharides (MS) showed that NnAG was composed primarily of arabinofuranose (Araf), galactofuranose (Galf) and glucopyranose. (B) GC-MS identification and relative quantification of partially methylated and acetylated reduced derivatives of major monosaccharides, showed that NnAG shared most of the structural features of AG isolated from mycobacteria, except for the presence of Galf disubstituted at both the 5 and 6 positions in the same proportion as 5-linked Galf.

**Figure 3:**
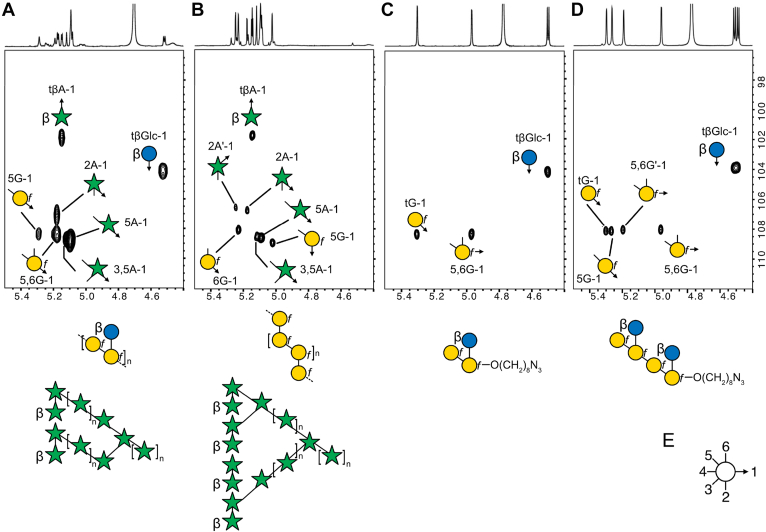
**Comparison of the main structural features of NnAG and MbAG.** Anomeric regions of the ^1^H–^13^C HSQC nuclear magnetic resonance spectra of NnAG (*A top panel*) and MbAG (*B top panel*) demonstrate that AG isolated from *N. nova* and *M. bovis* differ in the structure of both the arabinan and galactan moieties (*A* and *B lower panels*). In particular, the presence of an additional βGlc residue substituting a 5,6-αGal*f* residue in NnAG was established by comparison of ^1^H–^13^C HSQC and ^1^H–^13^C HMBC (Figs. 4 and S2) spectra. The exclusive presence of βGal*f*-5(βGlc*p*-6)βGal*f* epitope on NnAG was confirmed by comparison with the nuclear magnetic resonance spectra of synthetic compounds βGal*f*-5(βGlc*p*-6)βGal*f*-O(CH_2_)_8_N_3_ (NnAG1) (*C*) and [βGal*f*-5(βGlc*p*-6)βGal*f*]_2_-O(CH_2_)_8_N_3_ (NnAG2), the synthesis of which is described *below* (*D*). Symbol Nomenclature for Graphical Representations of Glycans (SNFG) was used to describe each monosaccharide and the linkages (C2, C3, C4, C5, C6) are represented according to (*E*) (39). All galactose residues (*G*, *yellow circles*) are β anomers; all arabinose residues (*A*, *green stars*) are α anomers, except for the terminal residues that are β anomers and labeled accordingly; all glucose residues (Glc, *blue circles*) are β anomers. Each signal was further labeled according to the i) linkage pattern of the associated monosaccharide as follows: tβGlc, terminal βGlc*p*; tG, terminal βGal*f*; 5G, βGal*f*; 5βGal*f*; 6G, 6βGal*f*; 5,6G, 5,6βGal*f*; tβA, terminal βAra; 2A, 2αAra; 5A, 5αAra; 3,5A, 3,5αAra and the ii) associated carbon/proton −1 to −6.

NnAG was further characterized by high field nuclear magnetic resonance (NMR) spectroscopy. Spin systems and chemical shifts obtained through a combination of homonuclear ^1^H–^1^H experiments (COSY, TOCSY) and heteronuclear ^1^H–^13^C experiments (HSQC, HSQC–TOCSY and HMBC) permitted the identification of seven types of monosaccharides in accordance with the composition and linkage analyses. Details were provided by ^1^H–^13^C HSQC experiments (Figs. 3; S1) and reported in Table 2. The polysaccharide was compared to the AG isolated from *M. bovis* BCG, which is known to be identical to AG from *M. tuberculosis* and all mycobacterial species studies reported to date (22). When compared with MbAG, NnAG exhibited marked differences in both the arabinan and galactan domains.

**Table 2:** ^1^H and^13^C chemical shifts of main monosaccharide residues of NnAG derived from ^1^H–^1^H TOCSY, ^1^H–^13^C HSQC, ^1^H–^13^C HSQC–TOCSY and ^1^H–^13^C HMBC NMR experiments

Residues	ppm	1	2	3	4	5	6/6′
tβGlc (tβGlc)	^1^H	4.526	3.350	3.503	3.406	3.478	3934/3730
	^13^C	104.12	74.37	76.95	70.8	77.12	61.87
βAra (tβA)	^1^H	5.148	4.157	4.062	3.913	8804/3.689	
	^13^C	101.86	77.45	75.41	83.32	64.11	
2αAra (2A)	^1^H	5.178	4.211	4.141	4.087	3.866/3.748	
	^13^C	107.0	88.16	76.03	84.03	61.89	
5αAra (5A)	^1^H	5.097	4.14	4.018	ND	3.894/3.807	
	^13^C	108.77	82.00	77.97	ND	68.26	
3,5αAra (3,5A)	^1^H	5.121	4.296	4.089	ND	3.915/3.826	
	^13^C	108.73	80.41	83.6	ND	67.63	
5βGal*f* (5G)	^1^H	5.292	4.14	4.107	4.226	3.962	3.808
	^13^C	108.37	82.70	77.86	83.61	76.78	62.43
5,6βGal*f* (5,6G)	^1^H	5.179	4.14	4.153	ND	4.518	4137/3896
	^13^C	108.41	82.5	82.63	ND	75.18	71.18

**Figure 4:**
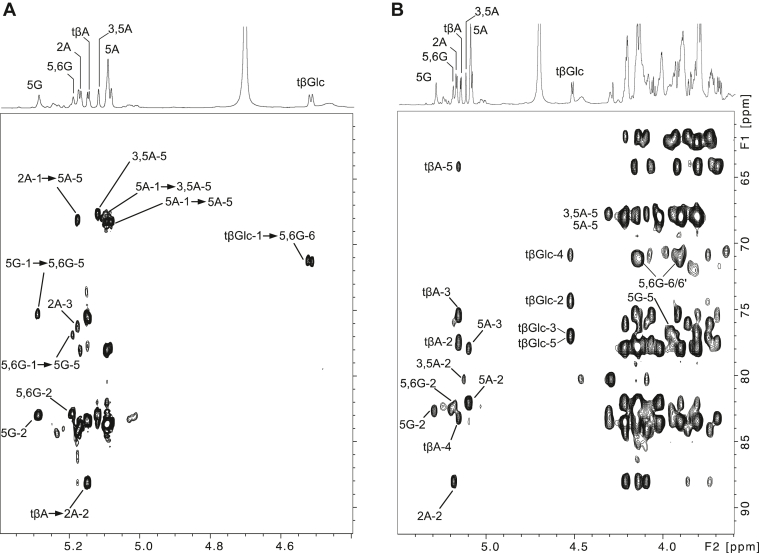
**Identification of the linkage pattern of arabinan and galactan moieties of NnAG.** Comparison of the ^1^H–^13^C HMBC (*A*) and ^1^H–^13^C HSQC–TOCSY (*B*) spectra of NnAG demonstrated that the tβGlc residue is linked to the C6 position of a 5,6-βGal*f* (5,6G) residue. This analysis also demonstrated that the 2αAra residue exclusively substitutes the C5 position of the arabinan chain contrary to MbAG in which Ara residues can be di-substituted at the C3 and C5 positions. In the ^1^H–^13^C HMBC spectrum, the ^3^*J*_H,C_ cross signals are represented as *arrows* between two residues.

Notably, although NnAG and MbAG share common features such as tβAra, 2αAra, 3αAra, 5αAra and 3,5αAra, NnAG contains a single signal associated with 2αAra (2A) whereas MbAG contains two (2A and 2A′). The ^1^H–^13^C HMBC spectrum of NnAG showed a single intense ^3^*J*_H,C_ connection between 2αAra-H1 at 5.178 ppm and 5αAra-C5 at 68.26 ppm (Fig. 4). In contrast, the ^1^H–^13^C HMBC spectrum of MbAG showed that the two 2αAra-H1 had differential ^3^*J*_H,C_ connections with 3,5αAra-C3 and 3,5αAra-C5 (data not shown), demonstrating that NnAG lacks the tβAra-2αAra-3αAra branch that is present in the Ara capping of MbAG as depicted in Figure 3, making the arabinan domain simpler. NMR experiments confirmed the presence of 5βGal*f* (5G) at δ ^1^H/^13^C 5.292/108.37 and 5,6βGal*f* (5,6G) at δ ^1^H/^13^C 5.179/108.41 in NnAG (Fig. 3*A*) instead of the alternating 5βGal*f* (5G) and 6βGal*f* (6G) observed in MbAG (Fig. 3*B*). The ^1^H–^13^C HMBC spectrum established that 5βGal*f* and 5,6βGal*f* were connected to each other through (1,5) linkages based on the strong 5βGal*f-*H1/5,6βGal*f*-C5 and 5,6βGal*f-*H1/5βGal*f*-C5 ^3^*J*_H,C_ cross signals (Fig. 4).

In addition, the ^1^H–^13^C HSQC spectrum of NnAG shows an intense signal at δ ^1^H/^13^C 4.526/104.12, which was further identified as a tβGlc*p* residue owing to its characteristic spin system (Table 2). A strong ^3^*J*_H,C_ cross signal between tβGlc*p*-H1 and 5,6βGal*f*-C6 (Fig. 4) unambiguously demonstrated that the βGlc residue substitutes the galactan moiety at the O6 position to generate a repetitive –[5βGal*f*-5(βGlc*p*-6)βGal*f*]_n_– motif. To confirm the structural attribution of the new Gal*f* backbone observed in NnAG, a series of oligosaccharide analog including the trisaccharide Gal*f*-5(βGlc*p*-6)βGal*f*-O(CH_2_)_8_N_3_ and the hexasaccharide [Gal*f*-5(βGlc*p*-6)βGal*f*]_2_-O(CH_2_)_8_N_3_ were synthesized (details below) and analyzed by NMR spectroscopy as shown in Figures S2 and S3. Of particular interest, the tβGlc*p* residues in both compounds showed identical NMR parameters to NnAG (Fig. 3) confirming its structure. The presence of a backbone composed of alternating 5βGal*f* and 5,6βGal*f* residues in NnAG was also confirmed by identical spin systems and chemical shifts compared to the internal 5βGal*f* and 5,6βGal*f* residues of the synthetic hexasaccharide (Fig. 3*D*).

Altogether, detailed structural analysis of the *N. nova* Fungitell-reactive fraction demonstrated that NnAG differs from mycobacterial AG in both arabinan and galactan domains. Of particular interest, the galactan domain is characterized by a β-glucose containing –[5βGal*f*-5(βGlc*p*-6)βGal*f*]_n_– repeating unit instead of a –[5βGal*f*-6βGal*f*]_n_– motif. Because the Fungitell assay detects BDG, which is composed of β-glucose residues, we hypothesize that this structural difference, in particular the β-Glc*p* side chains, was key to understanding the cross reactivity in the Fungitell test, which was further assessed as described below.

### Synthesis of repeating units of the NnAG galactan confirms the structure of the native polysaccharide and provides compounds for additional evaluation

**Figure 5:**
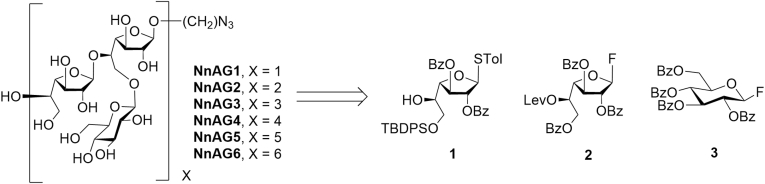
**Synthetic NnAG galactan fragments NnAG1–NnAG6 and the three monosaccharides (1–3) used for their preparation**.

To confirm the structure of the *N. nova* AG galactan as determined by NMR spectroscopy (see above) and to provide a series of structurally-defined compounds that could be evaluated in the Fungitell assay, six fragments of the polysaccharide (NnAG1–NnAG6, Fig. 5) were synthesized. The targets consist of 1 **to** 6 NnAG galactan repeating units, each functionalized with an azidooctyl aglycone to facilitate their future coupling to other species, *e.g.,* proteins for the generation of monoclonal antibodies. We envisioned that NnAG1–NnAG6 could be obtained from three monosaccharide building blocks: thioglycoside 1 and glycosyl fluorides 2 and 3 (Fig. 5).

**Figure 6:**
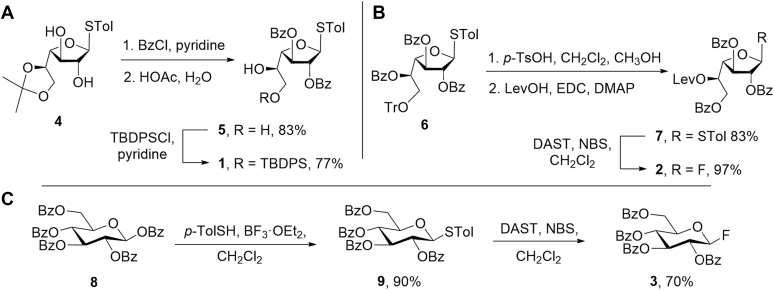
**Synthesis of building blocks 1–3**. *A*, Synthesis of 1, *B*, Synthesis of 2, *C,* Synthesis of 3.

**Figure 7:**
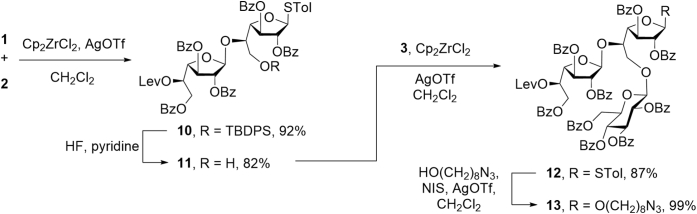
**Synthesis of trisaccharide 13**.

The synthesis of 1 to 3 is shown in Figure 6. Access to 1 started with the known thioglycoside 4 (24) (Fig. 7*A*). Benzoylation of the diol in 4 and acid hydrolysis of the isopropylidene ketal provided an 83% yield of 5, which was then treated with a limiting amount of *t*-butylchlorodiphenylsilane (TBDPSCl) in pyridine to generate 1 in 77% yield. Preparing 2 (Fig. 6*B*) started from 6 (25), which was first treated with *p*-toluenesulfonic acid (*p*-TsOH) in methanol and dichloromethane, triggering removal of the trityl either and subsequent migration of the O-5 benzoyl group to O-6. The intermediate alcohol formed in this process underwent Steglich esterification with levulinic acid affording an 83% yield of **7** over the two steps. Reaction of this thioglycoside with *N*,*N*-diethylaminosulfurtrifluoride and *N*-bromosuccinimide provided glycosyl fluoride 2 in 97% yield. Finally, the synthesis of 3 (Fig. 6*C*) was carried out starting from the perbenzoylated glucose derivative 8 (26) in two steps: conversion to thioglycoside 9 and then to the glycosyl fluoride under standard conditions to obtain the product in 63% overall yield.

These three monosaccharides were then used to generate a trisaccharide derivative (Fig. 7) that could be employed in the assembly of the target polysaccharide fragments. Initially, thioglycoside alcohol **1** and glycosyl fluoride 2 were coupled upon treatment with zirconocene dichloride and silver triflate (AgOTf) giving disaccharide 10 in 92% yields. Removal of the silyl ether protecting group afforded an 82% yield of disaccharide alcohol 11. Upon glycosylation of 11 with glycosyl fluoride 3, again promoted by zirconocene dichloride and AgOTf, an 87% yield of trisaccharide 12 was obtained. With this compound in hand, we explored its ability to act as a glycosyl donor *via* coupling with 8-azidooctanol using *N*-iodosuccinimde (NIS) and AgOTf as the promoters. This reaction cleanly provided the expected trisaccharide, 13 (99% yield). This result suggested that 12 could be efficiently used to access the larger fragments through an iterative process of selective cleavage of the levulinyl ester and glycosylation.

**Figure 8:**
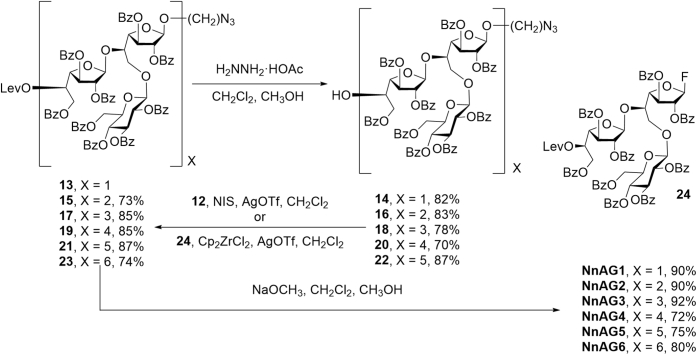
**Synthesis of NnAG1–NnAG6 *via* an iterative process of levulinate ester cleavage and glycosylation followed by deacylation**.

The application of the iterative process described above to the synthesis of NnAG1–NnAG6 is shown in Figure 8. Treatment of 13 with hydrazine acetate (NH_2_NH_2_·HOAc) selectively cleaved the levulinyl ester leading to an 82% yield of trisaccharide 14. Next, NIS/AgOTf-promoted glycosylation of 14 with 12 provided the expected hexasaccharide 15 in 73% yield. This cycle was applied one more time (15 → 16→17) in comparable yields; however, attempted glycosylation of nonasaccharide 18 with 12 failed to give the expected dodecasaccharide 19. Fortunately, the use of glycosyl fluoride 24 (Fig. 8, obtained in 99% yield by treatment of 12 with NIS and *N*,*N*-diethylaminosulfurtrifluoride) as the glycosyl donor gave 19 in 85% yield from 18. Further iteration of the cycle using 24 yielded the desired targets with 15 and 18 monosaccharide residues; 21 and 23, respectively. Access to NnAG1–NnAG6 was achieved in 72 to 92% yields by reaction of 13, 15, 17, 19, 21 or 23 with sodium methoxide in methanol.

As outlined above, the NMR data obtained from trisaccharide NnAG1 and dodecasaccharide NnAG2 agreed well with those obtained on the NnAG galactan domain, thus supporting the NMR structural assignments (Fig. 3).

### The β-glucose residues substituting the N. nova AG are responsible for Fungitell cross-reactivity

**Figure 9:**
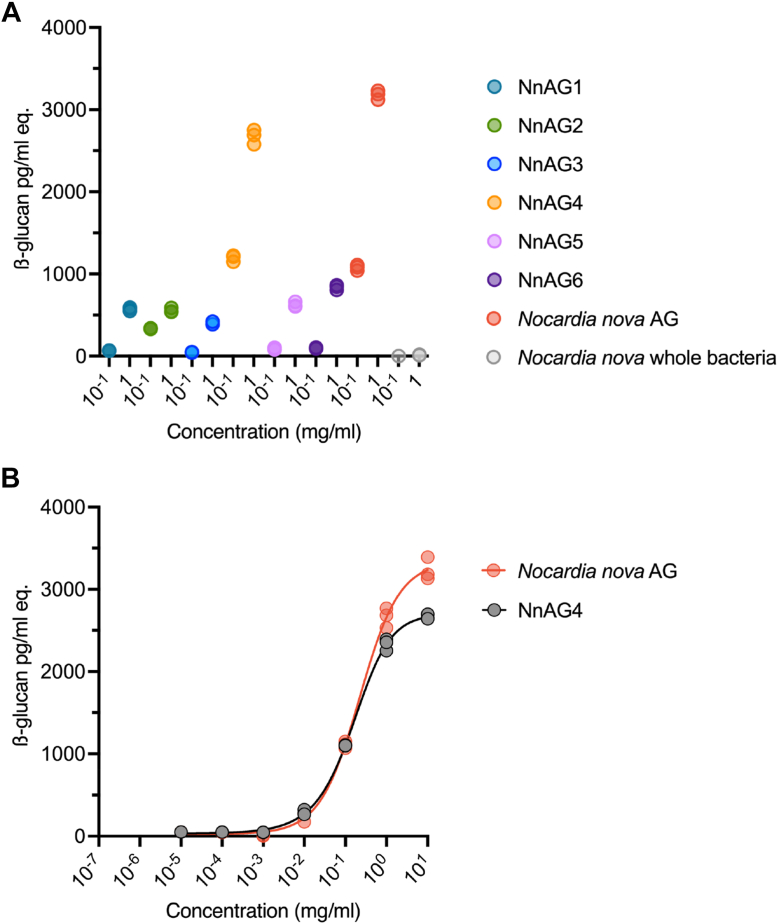
**Comparison of BDG reactivities of NnAG and synthetic oligosaccharides -5[(βGlcp-6)βGalf-5βGalf]-_n_.***A,* out of the six synthetic analogs (three replicates) of *N. nova* AG fragments, the dodecamer oligosaccharide (NnAG4) showed the highest reactivity to the β-(1,3)-D-glucan assay, equivalent to the natural AG at two different concentrations. *B,* concentration dependent assays showed a very similar trend for NnAG4 and natural *N. nova* AG (NnAG) (three replicates).

To confirm that the –[5βGal*f*-5(βGlc*p*-6)βGal*f*]_n_– motif observed in NnAG is indeed responsible the cross reactivity, the responsiveness of synthetic analog NnAG1–NnAG6 to Fungitell were evaluated in comparison with purified NnAG. Screening at two concentrations showed that all synthetic compounds were reactive at the highest concentration, but only NnAG4 showed significant reactivity equivalent to 1 ng of β-glucan/ml eq. at the lowest concentration and a very robust reactivity at the highest concentration, which are very similar to natural AG purified from *N. nova* (Fig. 9*A*). This was confirmed by comparing the dose-dependent reactivity of NnAG4 and natural *N. nova* AG, which showed very similar patterns (Fig. 9*B*).

In conclusion, the synthetic dodecasaccharide βGal*f*-5(βGlc*p*-6)βGal*f*-[5βGal*f*-5(βGlc*p*-6)βGal*f*]_3_-(CH_2_)_8_N_3_ (NnAG4), mimicking the galactan domain of NnAG, shows a reactivity level in the Fungitell assay comparable to intact NnAG. This demonstrates that the substitution of four βGlc*p* residues along the galactan backbone can trigger the activation of Fungitell, but that the polymerization degree of the synthetic compounds is critical for efficacy the activation.

## Discussion

The mortality rate of nocardiosis is high, estimated at 16.2% in European organ transplant recipients (27) and 17.5% in hematopoietic stem cell transplant cohorts (28). As such, there is an urgent need to identify methods for faster diagnosis and earlier treatment initiation in these vulnerable populations. These patients are also at risk to develop fungal infections justifying BDG testing in case of fever. Because various studies report positive BDG titers during *Nocardia* spp. infection (13, 14, 16, 18), discrepant experimental results may question this cross reactivity (13, 14, 18) and the reliability of this marker in evolutive cases of nocardiosis (29).

Detection of BDG in patient serum relies on its ability to activate the horseshoe crab coagulation cascade, which, in turn, cleaves an artificial substrate enabling colorimetric or turbidimetric detection (30). Several other BDG assays have been developed that differ in their threshold of detection, horseshoe crab species and detection method (9). Regardless of the assay, direct BDG dosage performed on whole bacteria cells were negative both in the present *N. nova* infection case using the Fungitell assay and in a reported *Nocardia farcinica* infection case using the Wako assay (Wako; Wako Pure Chemical Industries, Ltd) (14). We demonstrate here that a mechanical lysis step is required to detect a positive signal (641 pg/ml) and that a chemical cell wall extraction procedure made it possible possible to generate amounts of antigen that could be detected (560 pg/ml). The first step was necessary to uncover the reactive structure buried in the bacterial cell wall but was insufficient to reach high levels in patient sera (2600 pg/ml).

The genus *Nocardia* belongs to the Actinomycetes family, and is structurally related to mycobacteria (21). Its cell wall structure is characterized by a PG layer covalently connected *via* an AG polysaccharide to a lipid layer of mycolic acids. Owing to the similarity between *Nocardia* spp. and *Mycobacteria* spp., we followed a known cell wall fractionation protocol (20) to characterize the structure responsible for the Fungitell cross reactivity. The *N. nova* AG was identified as the key bacterial fraction responsible for the high apparent BDG rates. As previously described by Daffé et *al.*, *N. asteroides* and *N. brasiliensis*, AG structures do not have β-glucan architecture but rather possess a linear chain of galactofuranose with side-chain β-glucopyranose substitutions (21). Comparison of the GC/flame ionization detection analysis of glycosyl linkage for NnAG, compared to that published for *N. asteroides* and *N. brasiliensis*, showed a similar proportion of glucopyranose: 8.5% (NnAG) *versus* 10.2% (*N. asteroides* AG) and 10% (*N. brasiliensis* AG). Consistent with Daffé *et al.*, NMR experiments performed on *N. nova* AG assigned a β-D-configuration to the glucosyl residue and its terminal location on the AG chain. By testing purified extracts of AG in the Fungitell assay, NnAG proved to be more reactive than MbAG with a one-log shift in dose-response curves. The structure of NnAG galactan domain was confirmed by the chemical synthesis of structurally defined fragments, up to six repeating units (18 monosaccharide residues) in length, whose NMR spectra matched those of the native polysaccharide. It is noteworthy that AG isolated from *M*. *bovis* BCG, which has a galactan domain devoid of substituting βGlc(1,6) residues, retained a significant residual activity, albeit one to two log lower than NnAG. As demonstrated in the literature, factor G exhibits the highest degree of specificity towards linear (1,3)-β-D-glucans, and to a lesser extent towards branched β-D-glucans, provided that they contain 3-linkages. It has been established that certain common polysaccharides, including α-glucans, β-(1,4) glucans (*e.g.* cellulose), mannan, and PG, have the capacity to retain a residual activity that is four to five log lower than that exhibited by linear (1 → 3)-β-D-glucans (11). This finding suggests a certain degree of flexibility in the recognition process at the molecular level. Nevertheless, despite the observed low but significant cross-reactivity of mycobacterial AG *in vitro*, to the best of our knowledge, cases of positive BDG have never been reported in tuberculosis and non-tuberculosis mycobacteriosis patients. The absence of cross-reactivity may be attributable to a number of factors, including a reduced bacterial burden, a diminished efficacy in releasing AG into the bloodstream following lysis by immune cells in comparison to NnAG, or an inadequate activation of Factor G by AG *in vivo*.

To confirm that NnAG is indeed the endogenous molecule responsible for the BDG reactivity, the synthetic analogs (NnAG1–NnAG6) of the NnAG galactan, the purity and integrity of which were determined by high resolution NMR spectroscopy, were evaluated in the Fungitell assay. Remarkably, the synthetic analog NnAG4, a dodecasaccharide consisting of four repeating units exhibited a strong cross reactivity with BDG that was comparable to native NnAG, strongly suggesting that the substitution of the galactan domain by βGlc*p* residues was indeed responsible for the observed *in vivo* reactivity. These results provide, for the first time, direct biochemical proof that a purified cell wall component of *N. nova* cross reacts in assays designed to detect BDG. Surprisingly, although it was not negligible, the reactivity of longer polymers (NnAG5 and NnAG6) was lower that of NnAG4. This finding demonstrates that the Fungitell assay has a restricted specificity toward NnAG, possibly resulting from the in-solution conformation of the βGlc epitopes along the galactan backbone. Previous studies have demonstrated that in the case of (1,3)-β-D-glucans, the conformation, but not the molecular weight, appears be the dominant factor in Factor G activation potency, provided that the molecular weight is large enough to allow formation of the single helix conformation (12). However, the influence of structure, length and conformation of the glycan with the activation of Factor G has not, to date, been evaluated making it difficult to speculate about conformational changes observed in the different versions of the NnAG analogs. A rigorous investigation of the structure to function relationships of βGlc-substituted galactan is required to understand the specificity of the interaction with factor G and its subsequent activation.

Although our experiments demonstrate that cell-wall NnAG cross-reacts with BDG, we cannot completely eliminate the possibility of a cross-reactive compound being present in a loosely associated capsule-like layer. Mycobacteria are surrounded by an ill-defined capsular layer containing cell-wall derived polysaccharides, such as α-glucans, mannans and lipoarabinomannans (31), as well as other proteins, lipids and glycolipids. Although the existence of a capsule in mycobacteria has been postulated for decades, particularly through the work of Daffe and his collaborators (32), direct observation has proven difficult and has only recently been achieved through imaging whole-mount, plunge-frozen mycobacteria by cryoEM (33). However, the presence of AG fragments in this layer has never been documented, nor has the presence of a capsule in *nocardia* species been investigated. Therefore, we cannot rule out the presence of another cross-reactive, capsule-associated compound.

*Nocardia* spp. strains are known to interact with host macrophages during infection and, in some cases, to evade the phagocytosis process (34, 35). In an attempt to explain NnAG release leading to a BDG positive signal in patients, we performed macrophage infection experiments. High levels of apparent BDG were recorded after 12 h of incubation with values increasing according to the initial bacterial load (C2 > C1). These observations explain the high levels observed in the patient described here with an uncontrolled infection site (brain abscess). In our experiments, *in vitro* rates decreased after 24 h of incubation probably because of macrophage necrosis and bacterial degradation. These results may suggest a transitory phenomenon that can also be balanced by an unknown AG half-life in the host blood stream, and consequently explain the few Fungitell positive reported cases to date.

We demonstrate here that apparent BDG positivity in *N. nova* infection is not an incidental cross-reaction but relies on specific bacterial cell wall structures. Structural analysis of *N. nova* AG combined with macrophage infection experiments supported our hypothesis that Fungitell assay positivity in *N. nova* infections arises as a result of cross-reactivity with NnAG. Thus, an isolated apparent BDG elevation, without evidence of any fungal infection, should be considered as a potential marker of nocardiosis for clinicians and microbiologists. The availability of synthetic NnAG chemical analogs (NnAG1–NaAG6), lacking in any reactive contaminants, paves the way for the development of specific diagnosis tools (*e.g.,* monoclonal antibodies), that could be used in clinical settings.

## Experimental procedures

### Patient data and bacterial strain

The patient was diagnosed with a *Nocardia nova* infection at University Lille Hospital's Nephrology and Transplantation Department. Prior informed consent was obtained for the use of clinical data with the approval of the institutional Ethics Committee. The study complies with the Declaration of Helsinki principles. The infective strain was isolated from positive blood and lymph node culture. Bacterial identification was performed by MALDI TOF mass spectrometry (Bruker Daltonics). An accurate identification to the species level was given by a score value ≥ 1.9. The bacterial strain was sent to the French Referent Center for nocardiosis to confirm identification and perform antimicrobial susceptibility testing. For further experiments, the *N. nova* strain was stored at −80 °C in Müeller–Hinton medium supplemented with 10% glycerol until use.

### Fungitell assay procedures

The Fungitell assay (Associates of Cape Cod, E. Falmouth) was performed according to the manufacturer’s recommendations using patient sera; a cutoff value of 80 pg/ml apparent BDG was used as a positive result. In cases in which apparent BDG levels exceeded 500 pg/ml, samples were diluted and retested. For bacterial experiments, *N. nova* strain was suspended in glucan-free sterile saline, pelleted and washed according to the Koncan *et al.* procedure (13). Bead beating of the pellet was performed with glass beads during 70 s at 7000 rpm on MagNA Lyser (Roche, Boulogne-Billancourt). The pellet was then resuspended in 200 μl of glucan free saline and tested for fungitell positivity. Serum culture assays were performed in BDG-negative serum from a healthy donor of the Etablisse ment Français du Sang (EFS). For dose response experiments involving AG extracts of *N. nova, M. bovis* BCG and synthetic fragments, concentrations of the different fractions were standardized to 1 mg/ml and ranged from 0.01 μg/ml to 10 mg/ml to determine dose response relationship between AG concentration and Fungitell positivity. The interpretation of the Fungitell assay required adjustment for non-BDG samples. To address saturation issues, kinetic data, comprising both standards and samples, were analyzed to determine the slope of absorbance change at 405 nm over time (Figs. S4, S6, S8 and S10). Using BDG standards, a calibration curve was generated to depict the relationship between absorbance change per second and BDG concentration (abs405 nm vs. pg/ml). Subsequently, this calibration curve was employed to quantify the reactivity of samples in terms of BDG equivalents (pg/ml) (Figs. S5, S7, S9 and S11).

### Diagnosis of invasive fungal infection by mass spectrometry

As previously described*,* 1 μl of purified serum was spotted onto a MALDI plate followed by 1 μl of ionic liquid matrix preparation (36). Analysis was performed using an AB 4800 MALDI-TOF/TOF analyzer (Applied Biosystems/MDS Sciex) at fixed laser intensity for 1000 shots/spectrum. Signals were registered between *m/z* 300 and 800.

### Fractionation of nocardial and mycobacterial cell walls

The *N. nova* patient’s strain was cultivated in Sauton broth and the cell wall was fractionated essentially according to a procedure reported by Besra and coworkers (20) from a 1196 mg sample of lyophilized bacteria (Fig. 2*A*). Briefly, the bacterial pellet was rinsed with cold PBS and suspended in buffer PBS containing 2 mM NaCl, 2 mM MgCl_2_, and protease inhibitors (Halt Protease Inhibitor Cocktail w/o EDTA x100, Thermo Fisher Scientific) (1.0 g bacteria/ml) before lysis by French press (SLM Aminico French Pressure Cell Press FA-078) at a pressure of 1650 psi. The total lysate was centrifuged at 34,000*g* (JA-20 rotor, Beckman) at 4 °C for 30 min. The pellet (P1) was washed twice with PBS and freeze-dried. The supernatant (S1) was stored at −20 °C P1 was suspended in a 2% (v/v) solution of Triton X-100 by vortexing and then centrifuged in a glass tube at 24,000*g* (rotor JA-20, Beckman) at 4 °C for 30 min to obtain S2 and P2. P2 was washed twice with PBS and freeze-dried and then suspended in 20 ml of 2% SDS in PBS and heated to 95 °C for 1 h with vortexing every 15 min. After cooling, the suspension was centrifuged at 24,000*g* (JA-20 rotor, Beckman) at 4 °C for 30 min to obtain P3 and S3. This procedure was repeated twice. P3 was washed with ultrapure water, then with a mixture of water and acetone (20:80), followed by acetone, before being suspended in 20 ml of a 2% KOH solution in a mixture of toluene and methanol (50:50 vol/vol), and heated at reflux at 70 °C for 16 h. The solution was then cooled and centrifuged at 24,000*g* (JA-20 rotor, Beckman) at 20 °C for 20 min to yield P4 and S4. P4 was dried under nitrogen and weighed. Next, 25 ml of 2 M sodium hydroxide were added to P4 and the solution was heated at reflux at 80 °C for 16 h. After cooling, the solution was neutralized by the dropwise addition of 1 M hydrochloric acid centrifuged at 34,000*g* (JA-20 rotor, Beckman) at 20 °C for 30 min to yield P5 and S5. Fraction P5 was washed several times with ultrapure water to remove salts and lyophilized. Fraction S5 was filtered (0.45 μm, Millex syringe filter units, disposable, Durapore PVDF) and freeze-dried. The AG contained in S5 was dissolved in 10 ml of ultrapure water and dialysed (spectra/Por 6 standard regenerated cellulose dialysis membrane, Spectrum Laboratories Inc) against ultrapure water. The obtained AG was then lyophilized, weighed and stored at −20 °C. A similar procedure was adapted to purify AG from *M. bovis* BCG, which served as standard during the study.

### Composition analysis of isolated polysaccharides

Monosaccharides were identified and quantified as reduced and acetylated derivatives (alditol acetates) relative to internal *myo*-inositol standards. Polysaccharide samples (0.2–1 mg) were hydrolyzed with 4 M TFA at 110 °C for 3 h and then, after cooling dried. Samples were then placed in water and a few drops of 0.1 M NH_4_OH (pH 9) were added before the samples were reduced with sodium borohydride. Excess sodium borohydride was removed by addition of 10% acetic acid in methanol (MeOH), and the solution was dried under a stream of nitrogen. The drying process was repeated twice after addition of 10% acetic acid in MeOH (1 ml) and a further two times after addition of MeOH (1 ml). The residue was acetylated with 0.4 ml of acetic anhydride and 0.4 ml of pyridine at 100 °C for 1 h and was then dried under a stream of nitrogen with addition of toluene (1 ml) before being analyzed by GC-MS.

### Linkage analysis of isolated polysaccharides

Methylation was performed using the Ciucanu and Kerek procedure (37). The AG (0.5–1 mg) was dissolved in 1 ml of dimethyl sulfoxide. Freshly powdered NaOH (about 50 mg) was added and the mixture was stirred for 15 min. Methyl iodide (0.2 ml) was then added and the mixture stirred for another 2 h. The reaction was stopped by addition of 3 ml of 10% aqueous sodium thiosulfate (Na_2_S_2_O_3_). The permethylated product was extracted twice with CHCl_3_ (2 ml). The organic phase was washed five times with water (4 ml), filtered through a glass wool-plugged Pasteur pipette and the filtrate was evaporated. The product was hydrolyzed with 4 M TFA (110 °C, 3 h), cooled to room temperature, dried, reduced with sodium borodeuteride, and then peracetylated by incubation in acetic anhydride before being analyzed by GC-MS (see below). Methylated derivatives were identified using the Complex Carbohydrate Research Center partially methylated alditol acetates database (www.ccrc.uga.edu/specdb/ms/pmaa/pframe.html) and by comparison with authentic standards of methylation analysis of AG from mycobacteria (38).

### Gas chromatography of methylated alditol acetates

Gas chromatography (GC) was performed on a Trace GC Ultra system (Thermo Fisher Scientific) equipped with a NMTR-5MS capillary column (30 m × 0.25 mm) and a flame ionization detection unit using a temperature gradient from 170 °C to 250 °C at 5 °C·min^−1^. GC-MS was performed using a Trace GC Ultra system TSQ quantum GC detector (Thermo Fisher Scientific), equipped with a SILGEL1MS capillary column (30 m × 0.25 mm), and a temperature gradient from 170 °C to 230 °C at 3 °C min^−1^ then to 270 °C at 10 °C min^−1^.

### Nuclear magnetic resonance spectroscopy of isolated polysaccharides

Samples were solubilized in highly enriched deuterated water (D_2_O, 99.96% deuterium; EurisoTop, St-Aubin, France) and lyophilized. This process was repeated twice. Data were recorded on a 9.4-T spectrometer and an 18.8-T spectrometer (Infrastruture de Recherche-Très Hauts Champs-Résonance Magnétique Nucléaire, CNRS); at these field strengths, ^1^H resonate at 400 and 800 MHz, and ^13^C resonate at 100 and 201 MHz, respectively. As an internal standard, 1 μl of a solution of 2.5 μl of acetone in 10 ml of D_2_O was added to each sample. All pulse sequences were taken from the Bruker library of pulse programs and then optimized for each sample. Spectral widths were 12 and 200 ppm for the ^1^H and ^13^C observations, respectively. TOCSY was performed with various mixing times of 40 to 120 ms. Edited ^1^H–^13^C HSQC and HMBC spectra were recorded with 1536 data points for detection and 256 data points for indirect direction.

### Macrophage infection experiments

The THP-1 human pro-monocytic cell line (European Collection of Authenticated Cell Cultures, ECACC no. 88081201) was cultured at a density of 3 × 10ˆ5 cells/ml in RPMI-1640 medium (Roswell Park Memorial Institute Medium, Gibco), supplemented with 10% (v/v) decomplemented fetal calf serum (Dutcher), 2 mM L-glutamine and 20 μM β-mercaptoethanol. Cultures were kept under a humidified atmosphere containing 5% CO2 at 37 °C. To induce differentiation into a macrophage phenotype, THP-1 cells were treated with 20 nM phorbol-12-myristate-13-acetate (Sigma-Aldrich) for 72 h in sterile 24-well culture plates (*Nunclon Delta surface*, Thermo Fisher Scientific). PMA-activated THP-1 macrophages were then infected with two different concentrations of *Nocardia nova* (Multiplicity of Infection, MOI, 5:1 and 15:1) in RPMI-1640 medium devoid of serum for 6 h. The supernatants were then discarded, and the macrophages were cultured for an additional 12 h in serum-free RPMI-1640 medium supplemented with 20 μg/ml amikacin (amikacin sulfate, Sigma). Following incubation, the media were collected by centrifugation and subjected to Fungitell assay at a 1/1000 dilution. Control experiments included incubation of either bacteria alone or uninfected macrophages under identical conditions. Cells were tested negative for *Mycoplasma* spp.

### Synthesis of NnAG1–NnAG6

General methods, detailed procedures and spectroscopic data can be found in the Online Methods section.

## Data availability

All raw data supporting the findings of this study are available from the corresponding author, upon reasonable request.

## Supporting information

This article contains supporting information (24, 25, 26).

## Conflict of interest

The authors declare that they have no conflicts of interest with the contents of this article.

## Supporting information

Supporting information

**Abbreviations**
AG, arabinogalactan
AGP, arabinogalactan-peptidoglycan
AgOTf, silver triflate
Ara, arabinose
BDG, β-(1,3)-D-glucan
Gal, galactose
Gal*f*, galactofuranose
MeOH, methanol
NIS, *N*-iodosuccinimde
PG, peptidoglycan
