# Supplementary material for: Cell wall arabinogalactan is responsible for Fungitell cross reactivity in nocardiosis
Source: J Biol Chem. 2025 Nov 4;302(1):110900. doi: 10.1016/j.jbc.2025.110900 (PMC12794489; doi:10.1016/j.jbc.2025.110900)
Supplement: Supporting information [file mmc1.docx]

**Cell wall arabinogalactan is responsible for Fungitell® cross reactivity in nocardiosis**

**Supporting information**

Christophe Mariller ^1^, Pascal Letowski ^1^, Wei-Ting Chang ^2^, Todd L. Lowary ^2,3^, Marc Ulrich ^4^, Karine Faure ^5^, Séverine Loridant ^6^, Boualem Sendid ^6,7^, Frédéric Wallet^6^, Daniel Poulain^6^, Marc Hazzan ^4,10^, Marie Frimat ^4,8^, Yann Guerardel ^1,9^*, Marie Titecat ^6,10^*

^1^ Université de Lille, CNRS, UMR 8576 – UGSF - Unité de Glycobiologie Structurale et Fonctionnelle, F- 59000 Lille, France

^2^Institute of Biological Chemistry, Academia Sinica, Nangang, Taipei, 11529, Taiwan

^3^ Institute of Biochemical Sciences, National Taiwan University, Taipei, 10617, Taiwan

^4^ Univ. Lille, CHU Lille, Nephrology Department, F-59000, Lille, France

^5^ CHU Lille, Infectious Diseases Department, F-59000 Lille, France

^6^ CHU Lille, Institute of Microbiology, F-59000 Lille, France

^7^ Univ. Lille, CNRS UMR 8576, UGSF, Inserm U1285, CHU Lille, Laboratoire de Parasitologie-Mycologie, Lille, France.

^8^ Univ. Lille, Inserm, Institut Pasteur de Lille, U1167 - RID-AGE, F-59000 Lille, France

^9^ Institute for Glyco-core Research (iGCORE), Gifu University, Gifu, Japan

^10^ Université de Lille, INSERM, CHU Lille, U1286-INFINITE-Institute for Translational Research in Inflammation, Lille 59000, France

* **Correspondence should be addressed to:**

Dr Marie Titécat MD, PhD

Institute of Microbiology, Lille University Hospital

INFINITE-Institute for Translational Research in Inflammation, Lille 59000, France

Email: marie.titecat@chu-lille.fr

**Supplementary Figures**

SupFig 1 – Comparison of the main structural features of NnAG and MbAG.

SupFig 2 – Characterisation of βGalf-5(βGlcp-6)βGalf epitope from synthetic NnAG1

SupFig 3 – Characterisation of βGalf-5(βGlcp-6)βGalf internal and external epitopes from synthetic NnAG2.

SupFig 4 – Development of standard curve for Fungitell assay for samples shown in Fig.2B.

SupFig 5 – Fungitell assay for non-BDG samples (samples for Fig.2B)

SupFig 6 – Development of standard curve for Fungitell assay for samples in Fig.2C

SupFig 7 – Fungitell assay for non-BDG samples (samples for Fig.2C).

SupFig 8 – Development of standard curve for Fungitell assay for samples in Fig. 6A.

Sup Fig 9 – Fungitell assay for non-BDG samples (samples for Fig.6A).

Sup Fig 10 – Development of standard curve for Fungitell assay for samples in Fig. 6B

Sup Fig 11 – Fungitell assay for non-BDG samples (samples for Fig.6B).

**Supplementary Tables**

SupTable 1 - Patient characteristics

**Online methods**

Detailed procedures and data for the synthesis of NnAG1–NnAG6

**Sup Fig 1**

*SupFig 1 – Comparison of the main structural features of NnAG and MbAG*. Anomeric regions of the ^1^H–^13^C HSQC NMR spectra of NnAG (A top panel) and MbAG (B top panel) demonstrate that AG isolated from *N. nova* and *M. bovis* differ in the structure of both the arabinan and galactan moieties (A and B lower panels). In particular, the presence of an additional βGlc residue substituting a 5,6-αGal*f* residue in NnAG was established by comparison of ^1^H–^13^C HSQC and ^1^H–^13^C HMBC (Fig. 4 and SupFig. 1) spectra. The exclusive presence of βGal*f*-5(βGlc*p*-6)βGal*f* epitope on NnAG was confirmed by comparison with the NMR spectra of synthetic compounds βGal*f*-5(βGlc*p*-6)βGal*f*-O(CH_2_)_8_N_3_ (**NnAG1**) ©and [βGal*f*-5(βGlc*p*-6)βGal*f*]_2_-O(CH_2_)_8_N_3_ (**NnAG2**) (D).

**Sup Fig 2**


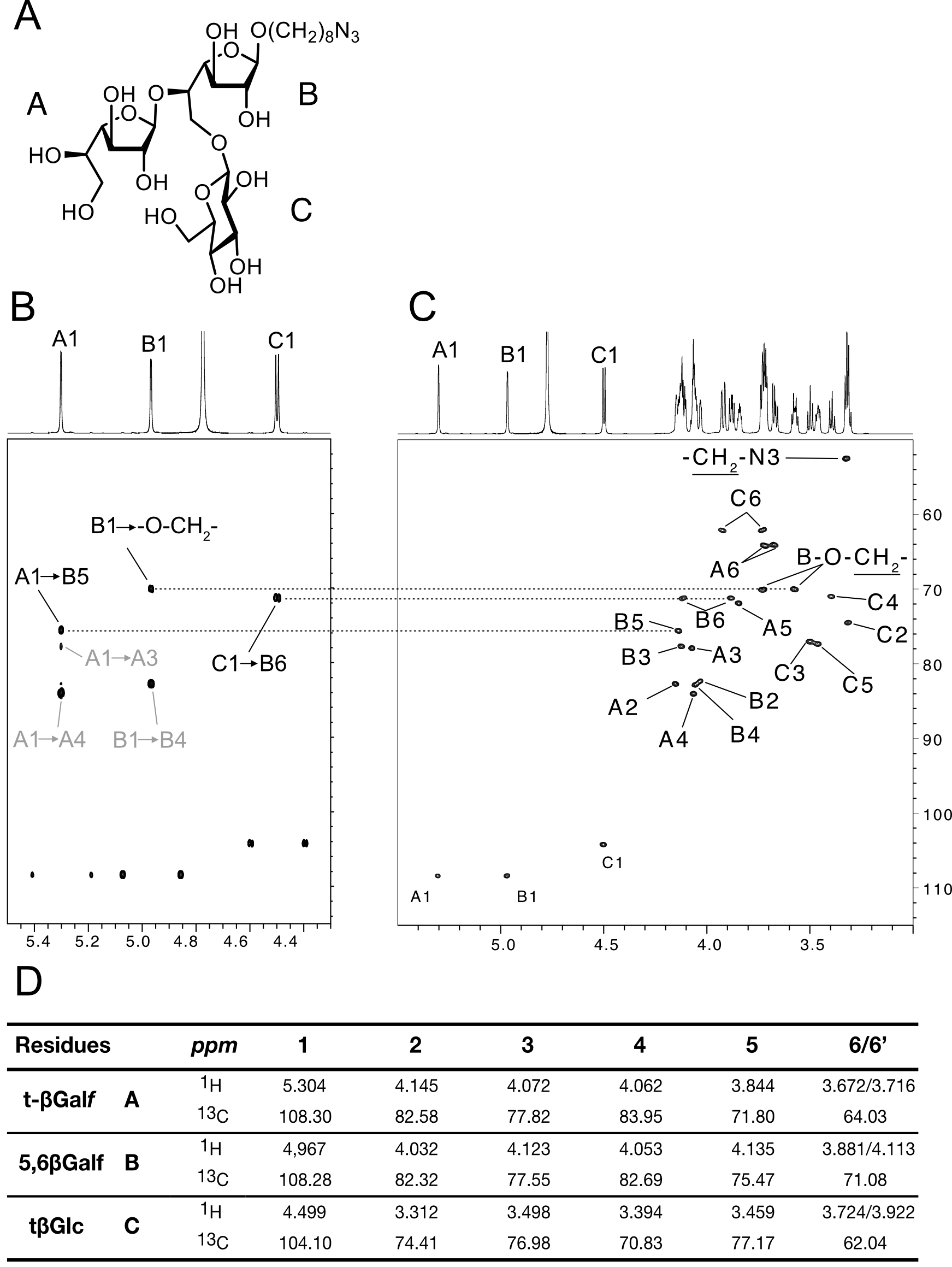


*SupFig 2 – Characterisation of βGalf-5(βGlcp-6)βGalf epitope from synthetic* ***NnAG1****.* (A) structure of the trisaccharide, (B) ^1^H–^13^C HMBC and (C) ^1^H–^13^C HSQC spectra showing the substitution of 5,6βGalf residue by a tβGlc residue in C6 position. (D) Full spin system of the trisaccharide deduced from ^1^H–^1^H COSY, ^1^H–^1^H TOCSY, ^1^H–^13^C HSQC and ^1^H–^13^C HSQC–TOCSY spectra. In (B), black labels represent inter-residues connections that demonstrates the sequence of the trisaccharide as represented in (A). Grey labels represent intra-residues connections.

**Sup Fig 3**


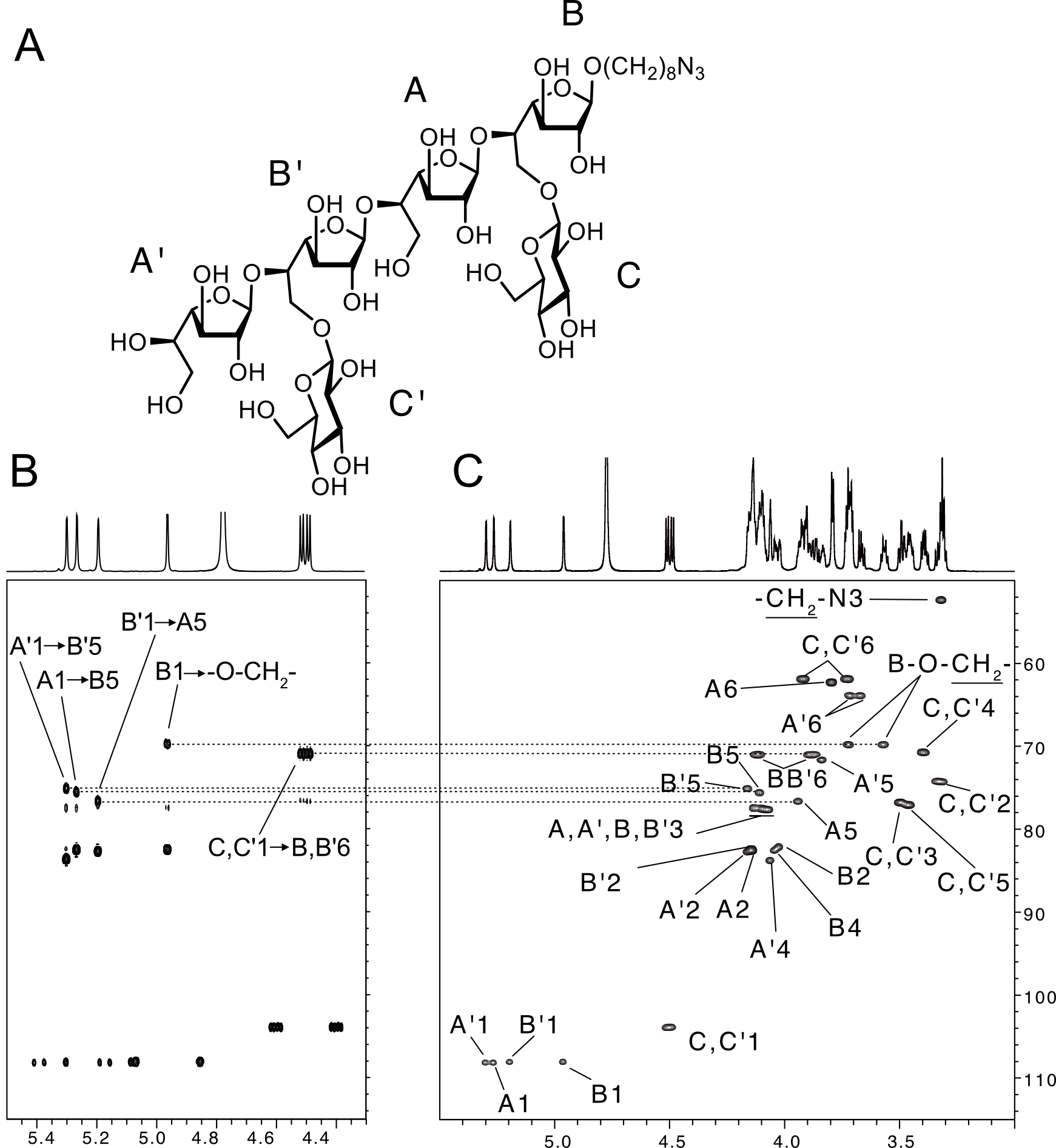


*SupFig 3 – Characterisation of βGalf-5(βGlcp-6)βGalf internal and external epitopes from synthetic* ***NnAG2****.* (A) Structure of the hexasaccharide, (B) ^1^H–^13^C HMBC and (C) ^1^H–^13^C HSQC spectra showing the substitution of 5,6βGalf residues B and B’ by tβGlc residues C and C’ in C6 position.

**Sup Fig 4**

*SupFig 4 – Development of standard curve for Fungitell assay for samples shown in Fig.2B.* (A) kinetic data obtained with BDG standards were extracted to determine the slope of absorbance at 405 nm over time, (B) calibration curve obtained used to quantify the reactivity of samples in terms of BDG equivalents (pg/ml).

**Sup Fig 5**

*SupFig 5 – Fungitell assay for non-BDG samples (samples for Fig.2B).* The slope of absorbance change at 405 nm over time is used with the calibration curve (SupFig. 3B) to obtain the reactivity of the sample in BDG equivalent.

**Sup Fig 6**

*SupFig 6 – Development of standard curve for Fungitell assay for samples in Fig.2C.* (A) kinetic data obtained with BDG standards were extracted to determine the slope of absorbance at 405 nm over time, (B) calibration curve obtained used to quantify the reactivity of samples in terms of BDG equivalents (pg/ml).

**Sup Fig 7**

*SupFig 7 – Fungitell assay for non-BDG samples (samples for Fig.2C).* The slope of absorbance change at 405 nm over time is used with the calibration curve (SupFig. 5B) to obtain the reactivity of the sample in BDG equivalent.

**Sup Fig 8**

*SupFig 8 – Development of standard curve for Fungitell assay for samples in Fig. 6A.* (A) kinetic data obtained with BDG standards were extracted to determine the slope of absorbance at 405 nm over time, (B) calibration curve obtained used to quantify the reactivity of samples in terms of BDG equivalents (pg/ml).

**Sup Fig 9**

*Sup Fig 9 – Fungitell assay for non-BDG samples (samples for Fig.6A).* The slope of absorbance change at 405 nm over time is used with the calibration curve (SupFig. 7B) to obtain the reactivity of the sample in BDG equivalent.

**Sup Fig 10**

*Sup Fig 10 – Development of standard curve for Fungitell assay for samples in Fig. 6B.* (A) kinetic data obtained with BDG standards were extracted to determine the slope of absorbance at 405 nm over time, (B) calibration curve obtained used to quantify the reactivity of samples in terms of BDG equivalents (pg/ml).

**Sup Fig 11**

*Sup Fig 11 – Fungitell assay for non-BDG samples (samples for Fig.6B).* The slope of absorbance change at 405 nm over time is used with the calibration curve (SupFig. 9B) to obtain the reactivity of the sample in BDG equivalent.

**Sup Table 1**

| **Comorbidities/events associated to increased risk for nocardiosis** | | |
| --- | --- | --- |
| Transplantation year | 2011 | |
| Increased IS in the previous year | + | |
| CMV disease in the previous year | + | |
| eGFR (mL/min/m^2^) | 40 | |
| Diabetes mellitus | – | |
| Autoimmune disease | anti-MPO vasculitis | |
| Neutropenia /VIH | –/– | |
| Chronic lung disease | – |  |
| Malignancy | – | |
| **Sites of *Nocardia* dissemination** |  | |
| primary site | cutaneous | |
| Secondary sites | axillar nod, cerebral abscess | |
| **Biological features at the admission** |  | |
| CRP (mg/dL) | 2.8 | |
| Leucocytes(10^9^/L) | 4340 | |
| CD4+ T lymphocytes (10^9^/L) | 11% | |
| hypogammaglobulinemia | + (5,5g/l) | |
| BDG peak (pg/mL) | 2000 | |
| **Microbiologic data** |  | |
| Candida serology | – | |
| Aspergillus serology | – | |
| Skin tissues |  | |
| *Mycelium/Candida (culture)* | –/– | |
| *Mycobacterium tuberculosis* | – | |
| *Bartonella (PCR/culture)* | –/– | |
| Axillar nod tissues |  | |
| *Nocardia species* | *N. nova* | |
| **Outcome** |  | |
| Interval first symptoms-diagnosis (d) | 52 | |
| interval admission-diagnosis (d) | 23 | |
| CRP (mg/dL) after ~7 days | 1.2 | |
| BDG (pg/mL) at ~14 days | 263 | |
| **Confounding factors of increased BDG** |  | |
| hemolysis/hyper-lipemia/-bilirubinemia | –/–/– | |
| antimicrobial agents in the 3 last mo | Fosfomycine prophylaxis (recurrent urinary tract infection) | |

BDG: β-(1,3)-D-Glucan; CMV: cytomegalovirus; CRP: C reactive protein; d: day; IS: immunosuppressive regimen; PCR: Polymerase chain reaction; TMP-SMX: trimethoprim-sulfamethoxazole.

*SupTable 1 - Patient characteristics*

**Online methods**

**Detailed procedures and data for the synthesis of NnAG1–NnAG6**

**General Methods.** Reactions were carried out in oven-dried glassware under an atmosphere of dry argon. All reagents used were purchased from commercial sources and were used without further purification unless noted. Oven-dried (135 °C) 4 Å molecular sieves were further activated by flame-drying under vacuum (0.5 mmHg) just prior to use. Dowex® 50W ion-exchange resin were washed by methanol for preparation. Solvents used in reactions were purified by successive passage through columns of alumina and copper under argon. Unless stated otherwise, all reactions were carried out at room temperature (rt) under a positive pressure of argon and were monitored by TLC on silica gel 60 F_254_. Spots were detected under UV light or by charring with cerium molybdate stain or *p*-anisaldehyde stain. Unless otherwise indicated, normal-phase column chromatography was performed on silica gel 60 (40–60 µm). The ratio between silica gel and crude product ranged from 150 to 50:1 (w/w). Size exclusion column chromatography was performed on HW-20. Reversed-phase column chromatography was performed on octadecyl silica (40–63 μm). In the processing of reaction mixtures, solutions of organic solvents were washed with equal volumes of aqueous solutions. Organic solutions were concentrated under vacuum at < 40˚C (bath). ^1^H NMR spectra were recorded at 500 or 600 MHz on a Bruker Avance 500 or 600 spectrometer, and chemical shifts are reported in ppm and referenced to either TMS (0.0 ppm, CDCl_3_) or HOD (4.78 ppm, D_2_O). ^13^C NMR spectra were recorded at 125 or 151 MHz, and ^13^C chemical shifts are reported in ppm referenced to internal CDCl_3_ (77.23 ppm, CDCl_3_) or external 2,2-dimethyl-2-silapentane-5-sulfonate sodium salt (DSS, –0.7 ppm, D_2_O). ^19^F NMR spectra were recorded at 470 or 564 MHz on a Bruker Avance 500 or 600 spectrometer. ^13^F chemical shifts are reported in ppm referenced to external CFCl_3_ (0 ppm, CDCl_3_). ^13^C NMR peak multiplicities, where reported, were inferred using either DEPT 135 or edited HSQC experiments. Where ^1^H and ^13^C NMR peak assignments are given, these were made unambiguously by a combination of ^1^H/^1^H COSY, ^1^H/^13^C HSQC, and ^1^H/^13^C HMBC experiments. Melting points were obtained using a Buchi melting point M-560 apparatus. Optical rotations were measured using Model 341 Polarimeter (PerkinElmer) at 22 ± 2 °C at the S4 sodium D line (589 nm) and are in units of deg·mL(dm·g)^–1^. High resolution mass spectra were measured by Mass Spectrometry facility of the Institute of Chemistry, Academia Sinica.

**8-Azidooctyl β-D-galactofuranosyl-(1**→**5)-[β-D-glucopyranosyl-(1**→**6)]-β-D-galactofuranoside (NnAG1)**. To a solution of trisaccharide **13** (15 mg, 9.5 μmol) in 3:1 CH_3_OH–CH_2_Cl_2_ (2 mL) was added sodium methoxide in CH_3_OH (0.1 M) dropwise until the pH of the reaction mixture was 12. After the solution was stirred at room temperature overnight, acid washed Dowex® 50W ion-exchange resin was added to adjust the pH of the mixture from alkaline to ~7. The resin was then removed by filtration and the filtrate was concentrated. The resulting residue was purified by reversed phase chromatography (18 mL gel, gradient from 10% CH_3_OH in H_2_O to 60% CH_3_OH in H_2_O). The product was evaporated to dryness, re-dissolved with H_2_O and lyophilized to afford **NnAG1** (4 mg, 90%) as a white foam. *R*_f_ 0.55 (*n*-propanol–H_2_O–AcOH, 10:2:1); [α]_D_ –101.0 (*c* 0.5, H_2_O); ^1^H NMR (500 MHz, D_2_O, δ_H_) 5.30 (d, *J* = 1.9 Hz, 1H, H-1’), 4.97 (d, *J* = 2.4 Hz, 1H, H-1), 4.50 (d, *J* = 7.9 Hz, 1H, H-1’’), 4.15–4.10 (m, 4H, H-3, H-2’, H-3’’, H-4’’), 4.09–4.01 (m, 4H, H-2, H-4, H-4’, H-2’’), 3.95–3.82 (m, 3H, H-5’, H-5’’, H-6b’’), 3.75–3.70 (m, 3H, H-6b, H-6b’, H-6a’’), 3.66 (dd, *J* = 11.7, 7.3 Hz, 1H, H-6a’), 3.57 (dt, *J* = 9.9, 6.5 Hz, 1H, H-6a), 3.52–3.43 (m, 2H, OCH_2_CH_2_, H-5), 3.39 (t, *J* = 9.4 Hz, 1H, OCH_2_CH_2_), 3.34–3.28 (m, 3H, H-3’, CH_2_N_3_), 1.60 (q, *J* = 7.0 Hz, 4H, octyl CH_2_), 1.42–1.30 (m, 8H, octyl CH_2_); ^13^C NMR (126 MHz, D_2_O, δ_C_) 107.0 (C-1, C-1’), 102.7 (C-1’’), 82.6 (C-4), 81.4 (C-4’), 81.3 (C-2), 80.9 (C-2’), 76.5 (C-5), 76.3 (C-3’’), 75.9 (C-2’’), 75.6 (C-5’’), 74.2 (C-3), 73.1 (C-3’), 70.5 (C-5’), 69.8 (C-4’’), 69.6 (C-6), 68.7 (OCH_2_CH_2_), 62.8 (C-6’), 60.7 (C-6’’), 51.2 (CH_2_N_3_), 28.5 (octyl CH_2_), 28.2 (octyl CH_2_), 28.1 (octyl CH_2_), 27.9 (octyl CH_2_), 25.8 (octyl CH_2_), 25.0 (octyl CH_2_); HRMS (ESI) Calcd for (M + Na) C_26_H_47_N_3_O_16_Na: 680.2849. Found 680.2848.

**8-Azidooctyl β-D-galactofuranosyl-(1→5)-[β-D-glucopyranosyl-(1→6)]-β-D-galactofuranosyl-(1**→**5)-β-D-galactofuranosyl-(1**→**5)-[β-D-glucopyranosyl-(1**→**6)]-β-D- galactofuranoside (NnAG2)**. Using the procedure described for the synthesis of **NnAG1**, hexasaccharide **15** (35 mg, 11.2 μmol) in 3:1 CH_3_OH–CH_2_Cl_2_ (2 mL) was converted to **NnAG2** (7 mg, 90%), which was obtained as a white foam. *R*_f_ 0.43 (*n*-propanol–H_2_O–AcOH, 10:2:1); [α]_D_ –95.0 (*c* 1.4, H_2_O); ^1^H NMR (600 MHz, D_2_O, δ_H_) 5.29 (d, *J* = 2.0 Hz, 1H), 5.26 (d, *J* = 1.8 Hz, 1H), 5.19 (d, *J* = 1.7 Hz, 1H), 4.96 (d, *J* = 2.3 Hz, 1H), 4.52–4.48 (m, 2H), 4.18–4.00 (m, 16H), 3.96–3.81 (m, 6H), 3.79–3.78 (m, 2H), 3.75–3.69 (m, 4H), 3.66 (dd, *J* = 11.7, 7.3 Hz, 1H), 3.56 (dt, *J* = 10.0, 6.5 Hz, 1H), 3.52–3.43 (m, 4H), 3.40–3.36 (m, 2H), 3.35–3.28 (m, 4H), 1.63–1.55 (m, 4H), 1.40–1.30 (m, 8H); ^13^C NMR (151 MHz, D_2_O, δ_C_) 107.02 (x 2), 106.98, 106.93, 102.8, 102.7, 82.6, 81.54, 81.47, 81.4, 81.3, 80.9, 76.5, 76.30, 76.25, 75.9, 75.6, 75.44, 74.40, 73.9, 73.1, 73.0, 70.4, 69.9, 69.8, 69.58, 69.55, 68.6, 62.7, 61.1, 60.71, 60.67, 51.2, 28.5, 28.2, 28.1, 27.9, 25.8, 25.0; HRMS (ESI) Calcd for (M + Na) C_44_H_77_N_3_O_31_Na: 1166.4433. Found 1166.4423.

**8-Azidooctyl β-D-galactofuranosyl-(1→5)-[β-D-glucopyranosyl-(1→6)]-β-D-galactofuranosyl-(1→5)-β-D-galactofuranosyl-(1→5)-[β-D-glucopyranosyl-(1→6)]-β-D-galactofuranosyl-(1→5)-β-D-galactofuranosyl-(1→5)-[β-D-glucopyranosyl-(1→6)]-β-D-galactofuranoside (NaAG3)**. Using the procedure described for the synthesis of **NnAG1**, nonasaccharide **17** (24 mg, 5.2 μmol) in 3:1 CH_3_OH–CH_2_Cl_2_ (0.6 mL) was converted to **NaAG3** (7 mg, 92%), which was obtained as a white foam. *R*_f_ 0.18 (*n*-propanol–H_2_O–AcOH, 10:2:1); [α]_D_ –71.4 (*c* 0.7, H_2_O); ^1^H NMR (500 MHz, D_2_O, δ_H_) 5.21 (d, *J* = 2.0 Hz, 1H), 5.18–5.17 (m, 2H), 5.10–5.09 (m, 2H), 4.87 (d, *J* = 2.4 Hz, 1H), 4.45–4.37 (m, 3H), 4.11–3.90 (m, 24H), 3.88–3.67 (m, 13H), 3.65–3.61 (m, 5H), 3.57 (dd, *J* = 11.7, 7.3 Hz, 1H), 3.48 (dt, *J* = 10.0, 6.5 Hz, 1H), 3.44–3.34 (m, 6H), 3.34–3.27 (m, 3H), 3.25–3.19 (m, 5H), 1.54–1.48 (m, 4H), 1.32–1.20 (m, 8H); ^13^C NMR (126 MHz, D_2_O, δ_C_) 107.02 (x 3), 106.97 (x 3), 102.8, 102.7 (x 2), 82.6, 81.53, 81.50, 81.47, 81.44, 81.39, 81.36, 81.34, 81.25, 80.9, 76.44, 76.38, 76.3, 76.2, 75.9, 75.6, 75.4, 75.3, 74.4, 74.0, 73.9, 73.1, 73.0, 70.4, 69.84, 69.81, 69.57, 69.55, 68.6, 62.7, 61.11, 61.06, 60.70, 60.67, 51.2, 28.5, 28.2, 28.1, 27.9, 25.8, 25.0; HRMS (ESI) Calcd for (M + Na) C_62_H_107_N_3_O_46_Na: 1652.6018. Found 1652.6015.

**8-azidooctyl β-D-galactofuranosyl-(1→5)-[β-D-glucopyranosyl-(1→6)]-β-D-galactofuranosyl-(1→5)-β-D-galactofuranosyl-(1→5)-[β-D-glucopyranosyl-(1→6)]-β-D-galactofuranosyl-(1→5)-β-D-galactofuranosyl-(1→5)-[β-D-glucopyranosyl-(1→6)]-β-D-galactofuranosyl-(1→5)-β-D-galactofuranosyl-(1→5)-[β-D-glucopyranosyl-(1→6)]-β-D-galactofuranoside (NnAG4)**. Using the procedure described for the synthesis of **NnAG1**, dodecsaccharide **19** (21 mg, 3.5 μmol) in 3:1 CH_3_OH–CH_2_Cl_2_ (1 mL) was converted to **NnAG4** (5 mg, 72%), which was obtained as a white foam. *R*_f_ 0.25 (*n*-propanol–H_2_O–AcOH, 6:2:1); [α]_D_ –42.0 (*c* 0.5, H_2_O); ^1^H NMR (500 MHz, D_2_O, δ_H_) 5.21 (d, *J* = 2.0 Hz, 1H), 5.18–5.17 (m, 3H), 5.10–5.09 (m, 3H), 4.87 (d, *J* = 2.4 Hz, 1H), 4.46–4.37 (m, 4H), 4.14–3.90 (m, 32H), 3.89–3.68 (m, 18H), 3.68–3.53 (m, 7H), 3.53–3.15 (m, 19H), 1.53–1.48 (m, 4H), 1.34–1.20 (m, 8H); ^13^C NMR (126 MHz, D_2_O, δ_C_) 107.02 (x 4), 106.95 (x 3), 106.9, 102.8 (x 3), 102.7, 81.52, 81.47, 81.44, 81.37, 81.28, 81.25, 76.44, 76.38, 76.3, 76.2, 75.9, 75.6, 73.1, 73.0, 69.84, 69.81, 69.57, 69.55, 62.7, 61.12, 61.07, 60.7, 51.2, 28.5, 28.2, 28.1, 27.9, 25.8, 25.0; HRMS (ESI) Calcd for (M + Na) C_80_H_137_N_3_O_61_Na: 2138.76026. Found 2138.75970.

**8-Azidooctyl β-D-galactofuranosyl-(1→5)-[β-D-glucopyranosyl-(1→6)]-β-D-galactofuranosyl-(1→5)-β-D-galactofuranosyl-(1→5)-[β-D-glucopyranosyl-(1→6)]-β-D-galactofuranosyl-(1→5)-β-D-galactofuranosyl-(1→5)-[β-D-glucopyranosyl-(1→6)]-β-D-galactofuranosyl-(1→5)-β-D-galactofuranosyl-(1→5)-[β-D-glucopyranosyl-(1→6)]-β-D-galactofuranosyl-(1→5)-β-D-galactofuranosyl-(1→5)-[β-D-glucopyranosyl-(1→6)]-β-D-galactofuranoside (NaAG5)**. Using the procedure described for the synthesis of **NaAG1**, pentadesaccharide **21** (25 mg, 3.4 μmol) in 3:1 CH_3_OH–CH_2_Cl_2_ (0.45 mL) was converted to **NaAG5** (5 mg, 75%), which was obtained as a white foam. *R*_f_ 0.23 (*n*-propanol–H_2_O–AcOH, 6:2:1); [α]_D_ –82.0 (*c* 0.5, H_2_O); ^1^H NMR (600 MHz, D_2_O, δ_H_) 5.30 (d, *J* = 2.0 Hz, 1H), 5.27–5.26 (m, 4H), 5.19–5.18 (m, 4H), 4.96 (d, *J* = 2.4 Hz, 1H), 4.53–4.47 (m, 5H), 4.17–4.00 (m, 42H), 3.95–3.92 (m, 6H), 3.92–3.85 (m, 8H), 3.80–3.79 (m, 8H), 3.75–3.69 (m, 8H), 3.51–3.44 (m, 10H), 3.41–3.36 (m, 5H), 3.36–3.29 (m, 7H), 1.64–1.54 (m, 4H), 1.38–1.29 (m, 8H); ^13^C NMR (151 MHz, D_2_O, δ_C_) 107.1, 107.03 (x 3), 107.02 (x 3), 107.01, 106.97, 106.9, 102.8 (x 4), 102.7, 82.6, 81.50, 81.45, 81.4, 81.3, 80.9, 76.5, 76.4, 76.3, 75.9, 75.6, 75.3, 74.4, 74.0, 73.9, 73.10, 73.05, 70.5, 69.9, 69.6, 68.7, 62.7, 61.7, 61.11, 61.07, 60.7, 51.2, 30.2, 28.5, 28.2, 28.1, 27.9, 25.8, 25.0; HRMS (ESI) Calcd for (M + Na) C_98_H_167_N_3_O_76_Na: 2624.9187. Found 2624.9204.

**8-Azidooctyl β-D-galactofuranosyl-(1→5)-[β-D-glucopyranosyl-(1→6)]-β-D-galactofuranosyl-(1→5)-β-D-galactofuranosyl-(1→5)-[β-D-glucopyranosyl-(1→6)]-β-D-galactofuranosyl-(1→5)-β-D-galactofuranosyl-(1→5)-[β-D-glucopyranosyl-(1→6)]-β-D-galactofuranosyl-(1→5)-β-D-galactofuranosyl-(1→5)-[β-D-glucopyranosyl-(1→6)]-β-D-galactofuranosyl-(1→5)-β-D-galactofuranosyl-(1→5)-[β-D-glucopyranosyl-(1→6)]-β-D-galactofuranosyl-(1→5)-β-D-galactofuranosyl-(1→5)-[β-D-glucopyranosyl-(1→6)]-β-D-galactofuranoside (NaAG6)**. Using the procedure described for the synthesis of **NaAG1**, octadecasaccharide **23** (17 mg, 1.9 μmol) in 3:1 CH_3_OH–CH_2_Cl_2_ (0.2 mL) was converted to **NaAG**6 (5 mg, 80%), which was obtained as a white foam. *R*_f_ 0.2 (*n*-propanol–H_2_O–AcOH, 6:2:1); [α]_D_ –50.0 (*c* 0.4, H_2_O); ^1^H NMR (500 MHz, D_2_O, δ_H_) 5.29 (d, *J* = 2.0 Hz, 1H), 5.27–5.26 (m, 5H), 5.19–5.18 (m, 5H), 4.96 (d, *J* = 2.4 Hz, 1H), 4.52–4.48 (m, 6H), 4.20–4.00 (m, 47H), 3.97–3.85 (m, 17H), 3.85–3.77 (m, 11H), 3.75–3.63 (m, 10H), 3.60–3.42 (m, 13H), 3.42–3.27 (m, 14H), 1.62–1.57 (m, 4H), 1.41–1.31 (m, 8H); ^13^C NMR (126 MHz, D_2_O, δ_C_) 107.029 (x 5), 107.026 (x 5), 106.95 (x 2), 102.8 (x 5), 102.7, 82.6, 81.47, 81.45, 81.34, 81.27, 81.2, 80.9, 76.44, 76.38, 76.3, 76.2, 75.9, 75.6, 75.4, 75.33, 75.28, 74.4, 74.0, 73.9, 73.1, 73.0, 70.4, 69.8, 69.5, 68.6, 62.7, 61.1, 60.7, 51.2, 28.5, 28.2, 28.1, 27.9, 25.8, 25.0; HRMS (ESI) Calcd for (M + Na) C_116_H_197_N_3_O_91_Na: 3111.0772 Found 3111.0783.

***p*-Tolyl 2,3-di-*O*-benzoyl-6-*O*-*t*-butyldiphenylsilyl-1-thio-β-D-galactofuranoside (1)**. To a solution of **5** (0.35 g, 0.7 mmol) in pyridine (1.2 mL) and CH_2_Cl_2_ (5.8 mL) at 0 °C, *t*-butyldiphenylsilyl chloride (1.1 mL, 4.2 mmol) was added dropwise. The solution was then stirred overnight while warming to room temperature before CH_3_OH (5 mL) was added. After stirring for 30 min, the solution was poured into a satd aq NaHCO_3_ soln (50 mL) and then extracted with CH_2_Cl_2_ (50 mL). The organic layer was washed with brine (15 mL), dried over Na_2_SO_4_, filtered and the filtrate was concentrated. The syrupy residue was purified by chromatography (hexanes–EtOAc, 7:1) to afford **1** (0.29 g, 77%) as a thick syrup. *R*_f_ 0.1 (hexane–EtOAc, 10:1); [α]_D_ –27.9 (*c* 1.76, CHCl_3_); ^1^H NMR (600 MHz, CDCl_3_, δ_H_) δ 8.16–8.10 (m, 2H, ArH), 8.07–8.00 (m, 2H, ArH), 7.67–7.65 (m, 4H, ArH), 7.64–7.54 (m, 2H, ArH), 7.49 (app t, *J* = 7.8 Hz, 2H, ArH), 7.46–7.36 (m, 6H, ArH), 7.34 (app t, *J* = 7.4 Hz, 4H, ArH), 7.08 (d, *J* = 7.9 Hz, 2H, ArH), 5.73 (dd, *J* = 5.4, 1.8 Hz, 1H, H-3), 5.70 (d, *J* = 1.7 Hz, 1H, H-1), 5.68 (app t, *J* = 1.8 Hz, 1H, H-2), 4.76 (dd, *J* = 5.4, 2.4 Hz, 1H, H-4), 4.21–4.18 (m, 1H, H-5), 3.83 (dd, *J* = 10.2, 6.3 Hz, 1H, H-6a), 3.77 (dd, *J* = 10.2, 6.7 Hz, 1H, H-6b), 2.45 (d, *J* = 7.6 Hz, 1H, OH), 2.33 (s, 3H, ArCH_3_), 1.05 (s, 9H, (CH_3_)_3_C); ^13^C NMR (151 MHz, CDCl_3_, δ_C_) δ 165.7 (C=O), 165.3 (C=O), 138.1 (Ar), 135.6 (Ar), 133.6 (Ar), 133.2 (Ar), 133.10 (Ar), 133.05 (Ar), 130.0 (Ar), 129.9 (Ar), 129.80 (Ar), 129.75 (Ar), 129.2 (Ar), 129.1 (Ar), 128.6 (Ar), 128.5 (Ar), 127.8 (Ar), 91.7 (C-1), 82.0 (C-2, C-4), 78.1 (C-3), 70.8 (C-5), 64.7 (C-6), 26.8 (CH_3_)_3_C), 21.2 (ArCH_3_), 19.2 ((CH_3_)_3_C); HRMS (ESI) Calcd for (M + Na) C_43_H_44_NaO_7_SSi: 755.2469. Found 755.2464.

**2,3,6-Tri-*O*-benzoyl-5-*O*-levulinoyl-β-D-galactofuranosyl fluoride (2)**. To a solution of 7 (6.3g, 9.0 mmol) in CH_2_Cl_2_ (213 mL) at 0 °C was added *N*,*N*-diethylamino sulfur trifluoride (4.3 mL, 32.5 mmol) followed by *N*-bromosuccinimide (5.68 g, 31.9 mmol). The reaction mixture was stirred overnight at room temperature and then CH_3_OH was added. The reaction mixture was diluted with CH_2_Cl_2_, and washed with satd aq NaHCO_3_ soln before the organic layer was separated. The aqueous layer was extracted again with CH_2_Cl_2_ and the combined organic layers were dried over Na_2_SO_4_. The solution was filtered and the filtrate was concentrated. The resulting residue was purified by chromatography (hexanes–EtOAc, 5:2) to afford **2** (5.3 g, 97%) as a thick syrup. *R*_f_ 0.2 (hexane–EtOAc, 2:1); [α]_D_ +28.2 (*c* 3.4, CHCl_3_); ^1^H NMR (600 MHz, CDCl_3_, δ_H_) δ 8.12–8.07 (m, 2H, ArH), 8.07–8.02 (m, 2H, ArH), 7.99–7.94 (m, 2H, ArH), 7.65–7.56 (m, 2H, ArH), 7.54–7.52 (m, 1H, ArH), 7.50–7.41 (m, 4H, ArH), 7.41–7.34 (m, 2H, ArH), 6.01 (d, *J* = 58.3 Hz, 1H, H-1), 5.79 (app dt, *J* = 7.0, 4.5 Hz, 1H, H-5), 5.68 (dd, *J* = 6.4, 1.1 Hz, 1H, H-2), 5.59 (dd, *J* = 4.1 Hz, 1.1 Hz, 1H, H-3), 4.82–4.76 (m, 1H, H-4), 4.71 (dd, *J* = 12.0, 4.5 Hz, 1H, H-6a), 4.57 (dd, *J* = 12.0, 7.0 Hz, 1H, H-6b), 2.86–2.38 (m, 4H, Lev CH_2_), 2.07 (s, 3H, Lev CH_3_); ^13^C NMR (151 MHz, CDCl_3_, δ_C_) 205.9 (Lev ketone C=O) ), 171.9 (Lev ester C=O) ), 166.0 (Ar), 133.9 (Ar), 133.8 (Ar), 133.2 (Ar), 130.0 (Ar), 129.8 (Ar), 129.5 (Ar), 128.7 (Ar), 128.5 (Ar), 128.4 (Ar), 112.3 (d, *J* = 226.5 Hz, C-1), 84.6 (C-4), 80.6 (d, *J* = 39.9 Hz, C-2), 76.1 (C-3), 70.0 (C-5), 63.0 (C-6), 37.9 (Lev CH_2_), 29.7 (Lev CH_3_), 28.0 (Lev CH_2_); ^19^F NMR (565 MHz, CDCl_3_, δ_F_) –125.36; HRMS (ESI) Calcd for (M + Na) C_32_H_29_FNaO_10_: 615.1637. Found 615.1638.

**2,3,4,6-Tetra-*O*-benzoyl-β-D-glucopyranosyl fluoride (3)**. To a solution of thioglycoside 9 (4.71 g, 6.7 mmol) in CH_2_Cl_2_ (71 mL) at 0 °C was added *N*,*N*-diethylaminosulfur trifluoride (2.4 mL, 18.9 mmol) followed by *N*-bromosuccinimide (3.2 g, 18.0 mmol). The solution was stirred at room temperature overnight and then neutralized by the addition of satd aq NaHCO_3_ soln. The solution was extracted with CH_2_Cl_2_ (100 mL) and the CH_2_Cl_2_ layer was then washed with water (30 mL × 3). The separated CH_2_Cl_2_ layer was dried over Na_2_SO_4_, filtered and the filtrate was concentrated. The resulting residue was purified by chromatography (hexane–EtOAc, 5:1) to afford glycosyl fluoride 3 (2.78 g, 70%) as a colorless syrup. *R*_f_ 0.15 (hexane–EtOAc, 5:1); ^1^H NMR (600 MHz, CDCl_3_, δ_H_) 8.13–8.06 (m, 2H, ArH), 8.06–8.01 (m, 2H, ArH), 7.99–7.94 (m, 2H, ArH), 7.94–7.90 (m, 2H, ArH), 7.63–7.56 (m, 2H, ArH), 7.56–7.52 (m, 1H, ArH), 7.52–7.48 (m, 1H, ArH), 7.46–7.42 (m, 4H, ArH), 7.39–7.35 (m, 4H, ArH), 5.91–5.84 (m, 2H, H-3, H-4), 5.74 (dd, *J* = 51.5, 5.4 Hz, 1H, H-1), 5.63 (ddd, *J* = 9.6, 7.3, 5.4 Hz, 1H, H-2), 4.74 (dd, *J* = 12.2, 3.7 Hz, 1H, H-6a), 4.59 (dd, *J* = 12.2, 5.2 Hz, 1H, H-6b), 4.44 (ddd, *J* = 8.8, 5.1, 3.8 Hz, 1H, H-5); ^13^C NMR (151 MHz, CDCl_3_, δ_C_) 166.1 (C=O), 165.5 (C=O), 165.1 (C=O), 164.9 (C=O), 133.6 (Ar), 133.6 (Ar), 133.5 (Ar), 133.2 (Ar), 130.0 (Ar), 129.9 (Ar), 129.9 (Ar), 129.8 (Ar), 128.7 (Ar), 128.6 (Ar), 128.6 (Ar), 128.5 (Ar), 128.5 (Ar), 128.4 (Ar), 106.5 (d, *J* = 221.1 Hz, C-1), 72.1 (d, *J* = 3.0 Hz, C-5), 71.4 (d, *J* = 7.2 Hz, C-3), 71.3 (d, *J* = 16.4 Hz, C-2), 68.2 (C-4), 63.0 (C-6); ^19^F NMR (565 MHz, CDCl_3_, δ_F_) –135.19; HRMS (ESI) Calcd for (M + Na) C_34_H_27_FNaO_9_: 621.1531. Found 621.1527.

***p*-Tolyl 2,3-di-*O*-benzoyl-1-thio-β-D-galactofuranoside (5)**. Compound **4** ^33^ (0.235 g, 0.72 mmol) was dissolved in pyridine (1.2 mL) and the solution cooled to 0 °C before benzoyl chloride (0.8 mL, mmol) was added dropwise. The reaction mixture was warmed to room temperature and stirred for 2 h. Excess benzoyl chloride was quenched by the addition of chilled water (50 mL) and the mixture was extracted with CH_2_Cl_2_ (20 mL). The CH_2_Cl_2_ layer was washed with satd aq NaHCO_3_ soln (10 mL) and water (10 mL × 3). The separated CH_2_Cl_2_ layer was dried over Na_2_SO_4_, filtered and the filtrate was concentrated. The syrupy residue obtained was dissolved in 4:1 acetic acid–H_2_O (3.6 mL) and the solution was heated at 65–70 °C overnight. After cooling to rt, the reaction mixture was concentrated and the residue was purified by column chromatography (hexanes–EtOAc, 3:2) to afford **5** (0.29 g, 83% over two steps) as a thick syrup. *R*_f_ 0.1 (hexane–EtOAc, 2:1); [α]_D_ –92.9 (*c* 1.13, CHCl_3_); ^1^H NMR (600 MHz, CDCl_3_, δ_H_) 8.16–8.09 (m, 2H, ArH), 8.08–8.03 (m, 2H, ArH), 7.65–7.55 (m, 2H, ArH), 7.53–7.41 (m, 6H, ArH), 7.14 (d, *J* = 7.9 Hz, 2H, ArH), 5.69 (s, 1H, H-1), 5.69 (app t, *J* = 1.5 Hz, 1H, H-3), 5.69–5.65 (m, 1H, H-2), 4.57 (dd, *J* = 4.8, 3.4 Hz, 1H, H-4), 4.20–4.13 (m, 1H, H-5), 3.89–3.75 (m, 2H, H-6 x 2), 2.78 (d, *J* = 7.7 Hz, 1H, OH), 2.34 (s, 3H, ArCH_3_); ^13^C NMR (151 MHz, CDCl_3_, δ_C_) δ 166.0 (C=O), 165.3 (C=O), 138.4 (Ar), 133.8 (Ar), 133.7 (Ar), 133.2 (Ar), 130.1 (Ar), 129.94 (Ar), 129.90 (Ar), 129.4 (Ar), 128.9 (Ar), 128.6 (Ar), 91.8 (C-1), 84.1 (C-4), 81.8 (C-2), 78.1 (C-3), 70.5 (C-5), 64.3 (C-6), 21.2 (ArCH_3_); HRMS (ESI) Calcd for (M + Na) C_27_H_26_NaO_7_S: 517.1291. Found 517.1282.

***p*-Tolyl 2,3,6-tri-*O*-benzoyl-5-*O*-levulinoyl-1-thio-β-D-galactofuranoside (7)**. Compound **6** ^21^ (0.5 g, 0.6 mmol) was dissolved in a solution of CH_2_Cl_2_–CH_3_OH (50:1, 12 mL) at 0 °C and *p*-toluenesulfonic acid (0.11 g, 0.6 mmol) was added. The reaction mixture was stirred overnight at rt. The mixture was diluted with EtOAc, poured into a satd aq NaHCO_3_ soln (20 mL) and extracted with EtOAc (40 mL). The organic layer was washed with water, dried over Na_2_SO_4_, filtered and the filtrate was concentrated to give the corresponding alcohol as a yellow syrup. This material was dissolved in pyridine (10 mL) and the solution was cooled to 0 °C before the addition of benzoyl chloride (1.1 mL, 10.1 mmol) dropwise. The reaction mixture was warmed to room temperature and stirred overnight. Excess benzoyl chloride was quenched by the addition of chilled water (30 mL) and the solution was extracted with CH_2_Cl_2_ (20 mL). The CH_2_Cl_2_ layer was washed with 10% aq copper sulfate soln (20 mL × 3) and then water (20 mL). The separated CH_2_Cl_2_ layer was dried over Na_2_SO_4_, filtered and the filtrate was concentrated. This material was then dissolved in CH_2_Cl_2_ (5 mL) and then levulinic acid (0.12 mL, 1.2 mmol), *N*-(3-dimethylaminopropyl)-*N’*-ethylcarbodiimide hydrochloride (0.28 g, 1.8 mmol), and 4-(dimethylamino) pyridine (0.36 g, 2.9 mmol) were added. The mixture was stirred for 4 h at rt. The reaction mixture washed with a satd aq NaHCO_3_ soln (5 mL) and brine (10 mL). The organic layer was then dried over Na_2_SO_4_, filtered and the filtrate was concentrated. The syrup residue was purified by column chromatography (hexanes–EtOAc, 2:1) to afford **7** (0.4 g, 83% over two steps) as a white foam. *R*_f_ 0.2 (hexane–EtOAc, 2:1); [α]_D_ –28.3 (*c* 2.3, CHCl_3_); ^1^H NMR (500 MHz, CDCl_3_, δ_H_) 8.11–8.04 (m, 4H, ArH), 8.00–7.96 (m, 2H, ArH), 7.62–7.38 (m, 11H, ArH), 7.12–7.08 (m, 2H, ArH), 5.78 (app dt, *J* = 7.0, 4.3 Hz, 1H, H-5), 5.72 (d, *J* = 1.9 Hz, 1H, H-1), 5.66 (app t, *J* = 1.9 Hz, 1H, H-2), 5.60 (dd, *J* = 4.6, 1.9 Hz, 1H, H-3), 4.79 (app t, *J* = 4.6 Hz, 1H, H-4), 4.66 (dd, *J* = 11.9, 4.3 Hz, 1H, H-6a), 4.54 (dd, *J* = 11.9, 7.0 Hz, 1H, H-6b), 2.71–2.52 (m, 4H, Lev CH_2_), 2.32 (s, 3H, ArCH_3_), 2.07 (s, 3H, Lev CH_3_); ^13^C NMR (151 MHz, CDCl_3_, δ_C_) 205.9 (Lev ketone C=O), 172.0 (Lev ester C=O), 166.0 (C=O), 165.4 (C=O), 165.3 (C=O), 138.3 (Ar), 133.6 (Ar), 133.1 (Ar), 133.1 (Ar), 130.1 (Ar), 129.93 (Ar), 129.87 (Ar), 129.8 (Ar), 129.6 (Ar), 128.9 (Ar), 128.6 (Ar), 128.4 (Ar), 91.4 (C-1), 82.0 (C-2), 81.1 (C-4), 77.4 (C-3), 69.9 (C-5), 63.2 (C-6), 37.9 (Lev CH_2_), 29.7 (Lev CH_3_), 28.0 (Lev CH_2_), 21.2 (ArCH_3_); HRMS (ESI) Calcd for (M + Na) C_39_H_36_NaO_10_S: 719.1921. Found 719.1929.

***p*-Tolyl 2,3,4,6-tetra-*O*-benzoyl-1-thio-β-D-glucopyranoside (9)**. To a solution of **8** ^22^(1.07 g, 1.5 mmol) in CH_2_Cl_2_ (2.8 mL) was added *p*-toluenethiol (0.21 g, 1.8 mmol). The mixture was cooled to 0 °C and then boron trifluoride diethyl etherate (2.1 mL, 1.8 mmol) was added dropwise. The solution was stirred at room temperature overnight and then neutralized by the addition of satd aq NaHCO_3_ soln dropwise. The solution was extracted with CH_2_Cl_2_ (100 mL) and the CH_2_Cl_2_ layer was washed with water (20 mL × 3). The separated CH_2_Cl_2_ layer was dried over Na_2_SO_4_, filtered and the filtrate was concentrated. The residue was recrystallized from EtOAc to give **9** as a white foam (460 mg). The mother liquor was concentrated and then purified by chromatography (hexane–EtOAc, 6:1) to give additional **9** (total yield = 0.97 g, 90%). *R*_f_ 0.15 (hexane–EtOAc, 7:1); mp 115 °C; ^1^H NMR (500 MHz, CDCl_3_, δ_H_) 8.04 (dd, *J* = 8.4, 1.4 Hz, 2H, ArH), 8.00–7.95 (m, 2H, ArH), 7.92–7.87 (m, 2H, ArH), 7.82–7.76 (m, 2H, ArH), 7.59 (ddt, *J* = 8.7, 7.3, 1.3 Hz, 1H, ArH), 7.55–7.31 (m, 11H, ArH), 7.29–7.23 (m, 2H, ArH), 6.98–6.89 (m, 2H, ArH), 5.90 (app t, *J* = 9.8, 9.7 Hz, 1H, H-3), 5.59 (app t, *J* = 9.8 Hz, 1H, H-4), 5.45 (app t, *J* = 9.7 Hz, 1H, H-2), 4.98 (d, *J* = 10.0 Hz, 1H, H-1), 4.68 (dd, *J* = 12.2, 2.8 Hz, 1H, H-6b), 4.48 (dd, *J* = 12.2, 5.7 Hz, 1H, H-6a), 4.17 (ddd, *J* = 10.0, 5.7, 2.8 Hz, 1H, H-5), 2.34 (s, 3H, ArCH_3_); ^13^C NMR (126 MHz, CDCl_3_, δ_C_) 166.1 (C=O), 165.8 (C=O), 165.2 (C=O), 165.0 (C=O), 138.6 (Ar), 133.9 (Ar), 133.5 (Ar), 133.3 (Ar), 133.2 (Ar), 133.1 (Ar), 129.88 (Ar), 129.86 (Ar), 129.8 (Ar), 129.6 (Ar), 129.3 (Ar), 128.8 (Ar), 128.7 (Ar), 128.4 (Ar), 128.3 (Ar), 127.6 (Ar), 86.2 (C-1), 76.3 (C-3), 74.2 (C-2), 70.5 (C-5), 69.4 (C-4), 63.1 (C-6), 21.1 (ArCH_3_); HRMS (ESI) Calcd for (M + Na) C_41_H_34_O_9_NaS: 725.1816. Found 725.1812.

***p*-Tolyl 2,3,6-tri-*O*-benzoyl-5-*O*-levulinoyl-β-D-galactofuranosyl-(1→5)-2,3-di-*O*-benzoyl-6-*O*-*t*-butyldiphenylsilyl-1-thio-β-D-galactofuranoside (10)**. A mixture of **1** (0.87 g, 1.2 mmol) and 4 Å molecular sieves (powder, 5.3 g) in CH_2_Cl_2_ (17 mL) was stirred at room temperature for 30 min under an Ar atmosphere, and then cooled to –10 °C before bis(cyclopentadienyl)zirconium (IV) dichloride (87 mg, 0.3 mmol) and silver trifluoromethanesulfonate (152 mg, 0.6 mmol) were added successively. After stirring for 10 min at –10 °C, glycosyl fluoride **2** (0.77 g, 1.3 mmol) in CH_2_Cl_2_ (2 mL) was added dropwise over 30 min. After stirring at –10 °C for 4 h, triethylamine was added and the resulting mixture was filtered through Celite. The filtrate was diluted with CH_2_Cl_2_ (30 mL) and washed with satd aq NaHCO_3_ soln. The organic layer was separated and the aqueous layer was extracted with CH_2_Cl_2_ (15 mL × 3). The combined organic layers were dried over Na_2_SO_4_, filtered, the filtrate was concentrated and the residue was purified by chromatography (hexanes–EtOAc, 5:2) to afford 10 (1.43 g, 92%) as a white foam. *R*_f_ 0.1 (hexane–EtOAc, 3:1); [α]_D_ –30.77 (*c* 0.78, CHCl_3_); ^1^H NMR (600 MHz, CDCl_3_, δ_H_) 8.04–7.98 (m, 4H, ArH), 7.94–7.90 (m, 2H, ArH), 7.90–7.85 (m, 4H, ArH), 7.63–7.62 (m, 4H, ArH), 7.60–7.53 (m, 2H, ArH), 7.49–7.36 (m, 9H, ArH), 7.36–7.26 (m, 10H, ArH), 7.21–7.18 (m, 2H, ArH), 7.00–6.98 (m, 2H, ArH), 5.76 (dd, *J* = 6.0, 2.8, 1H, H-3), 5.68 (dt, *J* = 7.8, 3.9 Hz, 1H, H-5’), 5.62 (d, *J* = 3.0 Hz, 1H, H-1), 5.61–5.58 (m, 2H, H-2, H-2’), 5.54 (app s, 1H, H-1’), 5.46 (dd, *J* = 5.2, 1.7 Hz, 1H, H-3’), 4.78 (dd, *J* = 5.2, 3.9 Hz, 1H, H-4’), 4.73–4.68 (m, 1H, H-4), 4.51 (dd, *J* = 12.0, 3.9 Hz, 1H, H-6a’), 4.46 (dd, *J* = 12.0, 7.8 Hz, 1H, H-6b’), 4.35 (dt , *J* = 6.5, 3.6 Hz, 1H, H-5), 3.97 (dd, *J* = 10.8, 6.5 Hz, 1H, H-6a), 3.93 (dd, *J* = 10.8, 3.6 Hz, 1H, H-6b), 2.65–2.34 (m, 4H, Lev CH_2_), 2.26 (s, 3H, ArCH_3_), 2.00 (s, 3H, Lev CH_3_), 0.98 (s, 9H, (CH_3_)_3_C); ^13^C NMR (151 MHz, CDCl_3_, δ_C_) δ 205.9 (Lev ketone C=O), 171.9 (Lev ester C=O), 165.9 (Ar), 165.5 (Ar), 165.40 (Ar), 165.36 (Ar), 165.0 (Ar), 137.9 (Ar), 135.5 (Ar), 133.45 (Ar), 133.43 (Ar), 133.36 (Ar), 133.3 (Ar), 132.90 (Ar), 132.87 (Ar), 132.82 (Ar), 132.79 (Ar), 130.0 (Ar), 129.9 (Ar), 129.8 (Ar), 129.73 (Ar), 129.69 (Ar), 129.60 (Ar), 129.55 (Ar), 129.1 (Ar), 129.0 (Ar), 128.86 (Ar), 128.85 (Ar), 128.5 (Ar), 128.4 (Ar), 128.2 (Ar), 127.8 (Ar), 127.7 (Ar), 105.3 (C-1’), 90.6 (C-1), 81.7 (C-2’), 81.6 (C-2), 81.5 (C-4’), 81.0 (C-4), 77.1 (C-3, C-3’), 75.9 (C-5), 70.0 (C-5’), 63.6 (C-6’), 63.4 (C-6), 37.9 (Lev CH_2_), 29.6 (Lev CH_3_), 27.9 (Lev CH_2_), 26.7 ((CH_3_)_3_C), 21.1 (ArCH_3_), 19.1 (CH_3_)_3_C); HRMS (ESI) Calcd for (M + Na) C_75_H_72_NaO_17_SSi: 1327.4151. Found 1327.4148.

***p*-Tolyl 2,3,6-tri-*O*-benzoyl-5-*O*-levulinoyl-β-D-galactofuranosyl-(1→5)-2,3-di-*O*-benzoyl-1-thio-β-D-galactofuranoside (11)**. Compound **10** (97 mg, 74 μmol) was dissolved in a solution of pyridine–THF (1:1, 0.8 mL) at 0 °C and 70% HF·pyridine (0.1 mL) was added dropwise. After the reaction was stirred for 15 h at rt, the mixture was diluted with EtOAc, poured into a satd aq NaHCO_3_ soln (10 mL) and extracted with EtOAc (30 mL). The organic layer was washed with water, dried over Na_2_SO_4_, filtered and the filtrate was concentrated. The resulting crude residue was purified by chromatography (hexane–EtOAc, 5:2) to afford alcohol **11** (65 mg, 82%) as a white foam. *R*_f_ 0.18 (hexane–EtOAc, 5:2); [α]_D_ –38.5 (*c* 0.52, CHCl_3_); ^1^H NMR (600 MHz, CDCl_3_, δ_H_) 8.12–8.07 (m, 2H, ArH), 8.04–8.02 (m, 2H, ArH), 7.99–7.98 (m, 2H, ArH), 7.95–7.93 (m, 2H, ArH), 7.89–7.87 (m, 2H, ArH), 7.62–7.54 (m, 2H, ArH), 7.52–7.46 (m, 3H, ArH), 7.46–7.41 (m, 6H, ArH), 7.40–7.35 (m, 2H, ArH), 7.35–7.31 (m, 2H, ArH), 7.25–7.21 (m, 2H, ArH), 7.10–7.05 (m, 2H, ArH), 5.80 (dd, *J* = 5.5, 2.2 Hz, 1H, H-3), 5.69 (d, *J* = 2.2 Hz, 1H, H-1), 5.65 (app dt, *J* = 7.7, 3.9 Hz, 1H, H-5’), 5.63–5.60 (m, 2H, H-1’, H-2), 5.56–5.55 (m, 2H, H-2’, H-4’), 4.77–4.72 (m, 1H, H-3’), 4.70 (dd, *J* = 5.5, 4.2 Hz, 1H, H-4), 4.48 (dd, *J* = 12.1, 3.9 Hz, 1H, H-6a’), 4.42 (dd, *J* = 12.1, 7.7 Hz, 1H, H-6b’), 4.33 (app dt, *J* = 6.0, 4.2 Hz, 1H, H-5), 4.00–3.93 (m, 2H, H-6a, H-6b), 2.69–2.48 (m, 4H, Lev CH_2_), 2.28 (s, 3H, ArCH_3_), 2.05 (s, 3H, Lev CH_3_); ^13^C NMR (151 MHz, CDCl_3_) δ 206.0 (Lev ketone C=O), 172.0 (Lev ester C=O), 166.00 (Ar), 165.95 (Ar), 165.50 (Ar), 165.47 (Ar), 165.4 (Ar), 138.4 (Ar), 133.7 (Ar), 133.60 (Ar), 133.56 (Ar), 133.4 (Ar), 133.2 (Ar), 133.01 (Ar), 130.03 (Ar), 130.0 (Ar), 129.9 (Ar), 129.82 (Ar), 129.78 (Ar), 129.7 (Ar), 129.6 (Ar), 129.3 (Ar), 128.92 (Ar), 128.90 (Ar), 128.87 (Ar), 128.8 (Ar), 128.6 (Ar), 128.5 (Ar), 128.3 (Ar), 105.3 (C-1’), 91.3 (C-1), 83.0 (C-4), 82.7 (C-4’), 81.7 (C-2, C-2’), 80.9 (C-3’), 77.4 (C-3), 75.9 (C-5), 69.9 (C-5’), 63.6 (C-6’), 62.2 (C-6), 37.9 (Lev CH_2_), 29.7 (Lev CH_3_), 28.0 (Lev CH_2_), 21.1 (ArCH_3_); HRMS (ESI) Calcd for (M + Na) C_59_H_54_NaO_17_S: 1089.2974. Found 1089.2979.

***p*-Tolyl 2,3,6-tri-*O*-benzoyl-5-*O*-levulinoyl-β-D-galactofuranosyl-(1→5)-[2,3,4,6-tetra-*O*-benzoyl-β-D-glucopyranosyl-(1→6)]-2,3-di-*O*-benzoyl-1-thio-β-D-galactofuranoside (12)**. A mixture of **11** (21 mg, 19.7 μmol) and 4 Å molecular sieves (powder, 0.

g) in CH_2_Cl_2_ (0.6 mL) was stirred at room temperature for 30 min under an Ar atmosphere and then cooled to –10 °C before bis(cyclopentadienyl)zirconium (IV) dichloride (11 mg, 1.81 mmol) and silver trifluoromethanesulfonate (19 mg, 34.0 μmol) were added successively. After stirring for 10 min at –10 °C, glycosyl fluoride **3** (30 mg, 37.6 μmol) in CH_2_Cl_2_ (0.2 mL) was added dropwise over 30 min. After stirring at –10 °C for 4 h, triethylamine was added and the resulting mixture was filtered through Celite. The filtrate was diluted with CH_2_Cl_2_ (2 mL), washed with satd aq NaHCO_3_ soln and the organic layer was separated and the aqueous layer was extracted with CH_2_Cl_2_ (2mL × 3). The combined organic layers were dried over Na_2_SO_4_, filtered, and the filtrate was concentrated. The residue was purified by chromatography (hexanes–acetone, 5:2) to afford 12 (27 mg, 87%) as a white foam. *R*_f_ 0.2 (hexanes–acetone, 5:2); [α]_D_ –24.0 (*c* 2.3, CHCl_3_); ^1^H NMR (500 MHz, CDCl_3_, δ_H_) δ 8.16–8.12 (m, 2H, ArH), 8.02–7.94 (m, 4H, ArH), 7.93–7.89 (m, 4H, ArH), 7.88–7.84 (m, 2H, ArH), 7.83–7.78 (m, 2H, ArH), 7.77–7.73 (m, 2H, ArH), 7.64–7.55 (m, 2H, ArH), 7.54–7.40 (m, 15H, ArH), 7.37–7.32 (m, 10H, ArH), 7.27–7.26 (m, 2H, ArH), 7.22–7.19 (m, 2H, ArH), 7.13–7.06 (m, 2H, ArH), 5.95 (app t, *J* = 9.6 Hz, 1H, H-3’’), 5.79–5.75 (m, 1H, H-3), 5.70–5.63 (m, 2H, H-4’’, H-5’), 5.60–5.59 (m, 2H, H-1, H-1’), 5.58–5.56 (m, 2H, H-2’’, H-2), 5.53 (app d, *J* = 1.5 Hz, 1H, H-2’), 5.46 (app d, *J* = 1.5, 4.7 Hz, 1H, H-3’), 5.07 (d, *J* = 7.8 Hz, 1H, H-1’’), 4.76 (app t, *J* = 4.7 Hz, 1H, H-4’), 4.70–4.64 (m, 1H, H-4), 4.59 (dd, *J* = 12.1, 3.2 Hz, 1H, H-6b’’), 4.54 (dd, *J* = 12.1, 3.7 Hz, 1H, H-6b’), 4.51–4.48 (m, 1H, H-5), 4.43 (dd, *J* = 12.0, 5.2 Hz, 1H, H-6a’’), 4.37 (dd, *J* = 12.0, 7.5 Hz, 1H, H-6a’), 4.26–4.20 (m, 2H, H-5’’, H-6b), 4.12 (dd, *J* = 10.9, 6.9 Hz, 1H, H-6a), 2.68–2.35 (m, 4H, Lev CH_2_), 2.30 (s, 3H, ArCH_3_), 2.03 (s, 3H, Lev CH_3_); ^13^C NMR (126 MHz, CDCl_3_, δ_C_) δ 205.9 (Lev ketone C=O), 171.2 (Lev ester C=O), 166.0 (Ar), 165.9 (Ar), 165.7 (Ar), 165.42 (Ar), 165.38 (Ar), 165.3 (Ar), 165.1 (Ar), 164.9 (Ar), 138.1 (Ar), 133.52 (Ar), 133.47 (Ar), 133.4 (Ar), 133.3 (Ar), 133.1 (Ar), 133.0 (Ar), 132.9 (Ar), 130.1 (Ar), 130.0 (Ar), 129.83 (Ar), 129.77 (Ar), 129.73 (Ar), 129.70 (Ar), 129.67 (Ar), 129.6 (Ar), 129.1 (Ar), 129.0 (Ar), 128.9 (Ar), 128.83 (Ar), 128.77 (Ar), 128.53 (Ar), 128.47 (Ar), 128.4 (Ar), 128.33 (Ar), 128.27 (Ar), 128.23 (Ar), 128.18 (Ar), 105.3 (C-1’), 100.6 (C-1’’), 90.9 (C-1), 81.9 (C-4), 81.7 (C-4’), 81.6 (C-2), 81.4 (C-2’), 77.2 (C-3), 77.3 (C-3’), 74.0 (C-5), 72.8 (C-3’’), 72.2 (C-5’’), 71.8 (C-2’’), 70.1 (C-5’), 69.8 (C-4’’), 67.9 (C-6), 63.4 (C-6’), 63.2 (C-6’’), 37.9 (Lev CH_2_), 29.6 (Lev CH_3_), 27.9 (Lev CH_2_), 21.1 (ArCH_3_); HRMS (ESI) Calcd for (M + Na) C_93_H_80_NaO_26_S: 1667.4550. Found 1667.4554.

**8-Azidooctyl 2,3,6-tri-*O*-benzoyl-5-*O*-levulinoyl-β-D-galactofuranosyl-(1→5)-[2,3,4,6-tetra-*O*-benzoyl-β-D-glucopyranosyl-(1→6)]-2,3-di-*O*-benzoyl-β-D-galactofuranoside (13)**. A mixture of **12** (0.15 g, 94 μmol), 8-azido-1-octanol (32 mg, 0.18 mmol) and 4 Å molecular sieves (powder, 1 g) in CH_2_Cl_2_ (3 mL) was stirred at room temperature for 30 min under an Ar atmosphere and then cooled to –10 °C before silver trifluoromethanesulfonate (24 mg, 93 μmol) and *N*-iodosuccinimide (27 mg, 93 μmol) were added successively. After stirring at –10 °C, for 4 h, triethylamine was added and the resulting mixture was filtered through Celite. The filtrate was diluted with CH_2_Cl_2_ (10 mL) and washed with satd aq NaHCO_3_ soln. The organic layer was separated and the aqueous layer was extracted with CH_2_Cl_2_ (10 mL × 3). The combined organic layers were dried over Na_2_SO_4_, filtered, the filtrate was concentrated and the residue was purified by chromatography (hexanes–acetone, 5:2) to afford 13 (0.16 g, 99%) as a white foam. *R*_f_ 0.15 (hexanes–acetone, 5:2); [α]_D_ –20.0 (*c* 0.15, CHCl_3_); ^1^H NMR (500 MHz, CDCl_3_, δ_H_) 8.15–8.09 (m, 2H, ArH), 8.00–7.91 (m, 8H, ArH), 7.91–7.86 (m, 2H, ArH), 7.85–7.80 (m, 2H, ArH), 7.80–7.76 (m, 2H, ArH), 7.76–7.72 (m, 2H, ArH), 7.62–7.57 (m, 1H, ArH), 7.57–7.51 (m, 1H, ArH), 7.51–7.29 (m, 21H, ArH), 7.27–7.18 (m, 2H, ArH), 7.14–7.07 (m, 2H, ArH), 5.93 (app t, *J* = 9.6 Hz, 1H, H-3”), 5.71–5.61 (m, 3H, H-3, H-5’, H-4”), 5.59 (app s, 1H, H-1’), 5.57–5.51 (m, 2H, H-2’, H-2”), 5.42 (dd, *J* = 5.1, 1.5 Hz, 1H, H-3’), 5.37 (app d, *J* = 1.6 Hz, 1H, H-2), 5.09 (app s, 1H, H-1), 5.06 (d, *J* = 7.8 Hz, 1H, H-1”), 4.79 (dd, *J* = 5.1, 4.3 Hz, 1H, H-4’), 4.63 (dd, *J* = 12.1, 3.5 Hz, 1H, H-6b”), 4.56 (dd, *J* = 12.1, 3.3 Hz, 1H, H-6b’), 4.50–4.35 (m, 4H, H-4, H-5, H-6a’, H-6a”), 4.29–4.16 (m, 2H, H-6b, H-5”), 4.10 (dd, *J* = 10.7, 7.0 Hz, 1H, H-6a), 3.63 (dt, *J* = 9.6, 6.6 Hz, 1H, OCH_2_CH_2_), 3.37 (dt, *J* = 9.6, 6.3 Hz, 1H, OCH_2_CH_2_), 3.21 (t, *J* = 7.0 Hz, 2H, CH_2_N_3_), 2.66–2.33 (m, 4H, Lev CH_2_), 2.00 (s, 3H, Lev CH_3_), 1.58–1.48 (m, 4H, octyl CH_2_), 1.34–1.26 (m, 8H, octyl CH_2_); ^13^C NMR (126 MHz, CDCl_3_, δ_C_) 205.9 (Lev ketone C=O), 171.9 (Lev ester C=O), 166.0 (Ar), 165.9 (Ar), 165.7 (Ar), 165.5 (Ar), 165.4 (Ar), 165.3 (Ar), 165.2 (Ar), 164.9 (Ar), 133.5 (Ar), 133.38 (Ar), 133.35 (Ar), 133.2 (Ar), 133.1 (Ar), 133.03 (Ar), 132.97 (Ar), 130.1 (Ar), 129.9 (Ar), 129.82 (Ar), 129.79 (Ar), 129.75 (Ar), 129.71 (Ar), 129.68 (Ar), 129.65 (Ar), 129.6 (Ar), 129.5 (Ar), 129.13 (Ar), 129.10 (Ar), 129.0 (Ar), 128.82 (Ar), 128.80 (Ar), 128.54 (Ar), 128.46 (Ar), 128.39 (Ar), 128.37 (Ar), 128.32 (Ar), 128.29 (Ar), 128.24 (Ar), 128.20 (Ar), 128.16 (Ar), 105.2 (C-1), 105.1 (C-1’), 100.6 (C-1”), 81.9 (C-4), 81.8 (C-4’), 81.6 (C-2), 81.5 (C-2’), 76.9 (C-3, C-3’), 73.7 (C-5), 72.9 (C-3”), 72.2 (C-2”), 71.8 (C-5”), 70.1 (C-5’), 69.8 (C-4”), 68.0 (C-6), 67.3 (OCH_2_CH_2_), 63.4 (C-6’), 63.2 (C-6”), 51.4 (CH_2_N_3_), 37.9 (Lev CH_2_), 29.6 (octyl CH_2_), 29.4 (octyl CH_2_), 29.3 (octyl CH_2_), 29.1 (octyl CH_2_), 28.8 (Lev CH_2_), 27.9 (Lev CH_3_), 26.7 (octyl CH_2_), 26.0 (octyl CH_2_); HRMS (ESI) Calcd for (M + Na) C_94_H_89_N_3_NaO_27_: 1714.5576. Found 1714.5575.

**8-Azidooctyl 2,3,6-tri-*O*-benzoyl-β-D-galactofuranosyl-(1→5)-[2,3,4,6-tetra-*O*-benzoyl-β-D-glucopyranosyl-(1→6)]-2,3-di-*O*-benzoyl-β-D-galactofuranoside (14)**. To a solution of **13** (150 mg, 88.6 μmol) and hydrazine acetate (13 mg, 14.1 μmol) were dissolved in CH_2_Cl_2_–CH_3_OH (1:2 mL). After stirring at room temperature for 4 h, the mixture was diluted with CH_2_Cl_2_ (10 mL) and washed with satd NaCl soln. The organic layer was separated and the aqueous layer was extracted with CH_2_Cl_2_ (10 mL × 3). The combined organic layers were dried over Na_2_SO_4_, filtered and the filtrate was concentrated. The residue was purified by chromatography (hexanes–acetone, 5:2) to afford 14 (0.12 g, 82%) as a white foam. *R*_f_ 0.23 (hexanes–acetone, 5:2); [α]_D_ –42.9 (*c* 0.07, CHCl_3_); ^1^H NMR (500 MHz, CDCl_3_, δ_H_) 8.12–8.03 (m, 2H, ArH), 8.01–7.97 (m, 4H, ArH), 7.94–7.89 (m, 6H, ArH), 7.82–7.71 (m, 6H, ArH), 7.62–7.57 (m, 1H, ArH), 7.56–7.28 (m, 20H, ArH), 7.25–7.21 (m, 2H, ArH), 7.19–7.16 (m, 2H, ArH), 7.12–7.05 (m, 2H, ArH), 5.94 (app t, *J* = 9.6 Hz, 1H, H-3”), 5.72–5.62 (m, 2H, H-3, H-4”), 5.62–5.56 (m, 2H, H-1’, H-2”), 5.55 (dd, *J* = 1.8, 0.7 Hz, 1H, H-2’), 5.52 (dd, *J* = 5.1, 1.8 Hz, 1H, H-3’), 5.36 (dd, *J* = 1.7, 0.6 Hz, 1H, H-2), 5.11–5.02 (m, 2H, H-1”, H-1), 4.68 (dd, *J* = 5.1, 3.0 Hz, 1H, H-4’), 4.59 (dd, *J* = 12.1, 3.3 Hz, 1H, H-6b), 4.53–4.42 (m, 3H, H-6a”, H-6b”, H-6b’), 4.40–4.33 (m, 2H, H-4, H-5), 4.27–4.24 (m, 2H, H-6a, H-5’), 4.21 (ddd, *J* = 9.1, 5.1, 3.3 Hz, 1H, H-5”), 4.10 (dd, *J* = 10.9, 7.3 Hz, 1H, H-6a’), 3.60 (dt, *J* = 9.5, 6.6 Hz, 1H, OCH_2_CH_2_), 3.36 (dt, *J* = 9.5, 6.3 Hz, 1H, OCH_2_CH_2_), 3.20 (t, *J* = 7.0 Hz, 2H, CH_2_N_3_), 2.63 (d, *J* = 9.0 Hz, 1H, OH), 1.52 (dd, *J* = 14.7, 7.6 Hz, 4H, octyl CH_2_), 1.35–1.22 (m, 8H, octyl CH_2_); ^13^C NMR (126 MHz, CDCl_3_, δ_C_) δ 166.1 (Ar), 165.8 (Ar), 165.6 (Ar), 133.5 (Ar), 133.40 (Ar), 133.35 (Ar), 133.2 (Ar), 133.14 (Ar), 133.07 (Ar), 132.9 (Ar), 130.1 (Ar), 129.9 (Ar), 129.8 (Ar), 129.70 (Ar), 129.68 (Ar), 129.6 (Ar), 129.5 (Ar), 129.1 (Ar), 129.0 (Ar), 128.9 (Ar), 128.8 (Ar), 128.6 (Ar), 128.5 (Ar), 128.38 (Ar), 128.35 (Ar), 128.32 (Ar), 128.26 (Ar), 128.2 (Ar), 128.1 (Ar), 105.3 (C-1), 105.0 (C-1’), 100.6 (C-1”), 83.2 (C-4), 82.1 (C-4’), 82.0 (C-2), 81.8 (C-2’), 78.2 (C-3’), 76.9 (C-3), 73.4 (C-5), 72.8 (C-3”), 72.2 (C-2”), 71.9 (C-5”), 69.8 (C-4”), 69.4 (C-5’), 69.1 (C-6), 67.3 (OCH_2_CH_2_), 66.2 (C-6’), 63.2 (C-6”), 51.4 (CH_2_N_3_), 29.4 (octyl CH_2_), 29.3 (octyl CH_2_), 29.1 (octyl CH_2_), 28.81 (octyl CH_2_), 26.7 (octyl CH_2_), 26.1 (octyl CH_2_); HRMS (ESI) Calcd for (M + Na) C_89_H_83_N_3_NaO_25_:1616.2508 Found 1616.2506.

**8-Azidooctyl 2,3,6-tri-*O*-benzoyl-5-*O*-levulinoyl-β-D-galactofuranosyl-(1→5)-[2,3,4,6-tetra-*O*-benzoyl-β-D-glucopyranosyl-(1→6)]-2,3-di-*O*-benzoyl-β-D-galactofuranosyl-(1→5)-2,3,6-tri-*O*-benzoyl-β-D-galactofuranosyl-(1→5)-[2,3,4,6-tetra-*O*-benzoyl-β-D-glucopyranosyl-(1→6)]-2,3-di-*O*-benzoyl-β-D-galactofuranoside (15)**. A mixture of alcohol **14** (50 mg, 31 μmol), thioglycoside **12** (62 mg, 38 μmol) and 4 Å molecular sieves (powder, 0.33 g) in CH_2_Cl_2_ (1.2 mL) was stirred at room temperature for 30 min and then the mixture as cooled to –5 °C before silver trifluoromethanesulfonate (17 mg, 65 μmol) and *N*-iodosuccinimide (16 mg, 63 μmol) were added successively. After stirring at –5 °C for 4 h, triethylamine was added and the resulting mixture was filtered through Celite. The filtrate was diluted with CH_2_Cl_2_ (10 mL), washed with satd aq NaHCO_3_ soln and the organic layer was separated. The aqueous layer was extracted with CH_2_Cl_2_ (10 mL × 3) and the combined organic layer was dried over Na_2_SO_4_, filtered and the filtrate was concentrated. The residue was purified by chromatography (hexanes–acetone, 2:1) to afford 15 (65 mg, 73%) as a white foam. *R*_f_ 0.1 (hexanes–acetone, 2:1); [α]_D_ –7.3 (*c* 3.2, CHCl_3_); ^1^H NMR (600 MHz, CDCl_3_, δ_H_) 8.12–8.03 (m, 4H), 7.98–7.91 (m, 6H), 7.91–7.83 (m, 11H), 7.82–7.62 (m, 16H), 7.56 (ddt, *J* = 7.9, 7.0, 1.3 Hz, 1H), 7.50–7.26 (m, 33H), 7.26–7.12 (m, 13H), 7.11–6.99 (m, 6H), 5.91 (app t, *J* = 9.6 Hz, 1H), 5.79 (app t, *J* = 9.6 Hz, 1H), 5.70–5.68 (m, *J* = 5.5 Hz, 2H), 5.67–5.65 (m, 2H), 5.63–5.61 (m, 2H), 5.59–5.58 (m, 3H), 5.55 (s, 1H), 5.54–5.49 (m, 2H), 5.49–5.45 (m, 1H), 5.43 (dd, *J* = 1.7, 0.6 Hz, 1H), 5.37–5.32 (m, 2H), 5.07 (s, 1H), 5.03 (d, *J* = 7.7 Hz, 1H), 4.83–4.81 (m, 2H), 4.67 (dd, *J* = 11.8, 3.8 Hz, 1H), 4.62 (app t, *J* = 4.7 Hz, 1H), 4.59 (dt, *J* = 7.0, 3.4 Hz, 1H), 4.54 (dd, *J* = 6.2, 3.1 Hz, 1H), 4.53–4.47 (m, 3H), 4.46–4.43 (m, 2H), 4.40–4.35 (m, 3H), 4.34–4.27 (m, 2H), 4.24 (dd, *J* = 10.6, 5.6 Hz, 1H), 4.19–4.16 (m, 2H), 4.10–4.03 (m, 2H), 3.95–3.92 (m, 1H), 3.60 (dt, *J* = 9.6, 6.6 Hz, 1H, OCH_2_CH_2_), 3.34 (dt, *J* = 9.6, 6.3 Hz, 1H, OCH_2_CH_2_), 3.19 (t, *J* = 7.0 Hz, 2H, CH_2_N_3_), 2.53–2.11 (m, 4H, Lev CH_2_), 1.93 (s, 3H, Lev CH_3_), 1.56–1.43 (m, 4H, octyl CH_2_), 1.34–1.21 (m, 8H, octyl CH_2_); ^13^C NMR (151 MHz, CDCl_3_, δ_C_) 205.9, 166.0, 165.9, 165.8, 165.7, 165.5, 165.4, 165.3, 165.1, 164.9, 164.8, 133.3, 133.2, 132.9, 132.8, 130.1, 130.0, 129.82, 129.76, 129.71, 129.69, 129.66, 129.60, 129.55, 129.5, 129.1, 128.98, 128.95, 128.92, 128.86, 128.85, 128.8, 128.7, 128.51, 128.46, 128.4, 128.30, 128.28, 128.21, 128.17, 128.11, 128.07, 105.8, 105.1, 105.0, 101.1, 100.5, 83.2, 82.1, 82.0, 81.8, 81.5, 77.8, 73.5, 73.0, 72.9, 72.2, 72.0, 71.8, 71.3, 70.1, 69.9, 69.5, 67.2, 65.0, 63.6, 63.3, 62.7, 51.4, 37.8, 29.6, 29.3, 29.1, 28.8, 27.8, 26.7, 26.0; HRMS (ESI) Calcd for (M + Na) C_175_H_155_N_3_NaO_51_: 3139.1428. Found 3139.1471.

**8-Azidooctyl 2,3,6-tri-*O*-benzoyl-β-D-galactofuranosyl-(1→5)-[2,3,4,6-tetra-*O*-benzoyl-β-D-glucopyranosyl-(1→6)]-2,3-di-*O*-benzoyl-β-D-galactofuranosyl-(1→5)-2,3,6-tri-*O*-benzoyl-β-D-galactofuranosyl-(1→5)-[2,3,4,6-tetra-*O*-benzoyl-β-D-glucopyranosyl-(1→6)]-2,3-di-*O*-benzoyl-β-D-galactofuranoside (16)**. Hexasaccharide **15** (200 mg, 64 μmol) and hydrazine acetate (12 mg, 0.12 mmol) were dissolved in CH_2_Cl_2_–CH_3_OH (0.7:1.4 mL). After stirring at room temperature for 4 h, the mixture was diluted with CH_2_Cl_2_ (10 mL) and washed with a satd NaCl aq soln. The organic layer was separated and the aqueous layer was extracted with CH_2_Cl_2_ (10 mL × 3). The combined organic layers were dried over Na_2_SO_4_, filtered, and the filtrate was concentrated. The residue was purified by chromatography (hexanes–EtOAc, 1:2) to afford 16 (0.16 g, 83%) as a white foam. *R*_f_ 0.49 (hexanes–EtOAc, 1:1); [α]_D_ –12.7 (*c* 0.71, CHCl_3_); ^1^H NMR (500 MHz, CDCl_3_, δ_H_) δ 8.07–8.02 (m, 4H), 7.99–7.95 (m, 8H), 7.92–7.87 (m, 8H), 7.84–7.78 (m, 6H), 7.77–7.70 (m, 8H), 7.68–7.65 (m, 2H), 7.62–7.57 (m, 1H), 7.53–7.48 (m, 4H), 7.48–7.40 (m, 10H), 7.40–7.31 (m, 17H), 7.28–7.24 (m, 9H), 7.24–7.04 (m, 13H), 5.93 (app t, *J* = 9.6 Hz, 1H), 5.86 (app t, *J* = 9.6 Hz, 1H), 5.73–5.71 (m, 3H), 5.68 (d, *J* = 9.7 Hz, 1H), 5.65–5.63 (m, 3H), 5.62–5.59 (m, 2H), 5.58–5.51 (m, 3H), 5.48–5.47 (m, 2H), 5.36 (d, *J* = 1.4 Hz, 1H), 5.09 (s, 1H), 5.05 (d, *J* = 7.8 Hz, 1H), 4.88 (d, *J* = 7.8 Hz, 1H), 4.83 (dd, *J* = 4.6, 3.1 Hz, 1H), 4.74–4.67 (m, 1H), 4.62–4.52 (m, 5H), 4.51–4.44 (m, 2H), 4.44–4.16 (m, 9H), 4.16–4.03 (m, 3H), 4.00 (dt, *J* = 9.9, 3.6 Hz, 1H), 3.67–3.58 (m, 1H, OCH_2_CH_2_), 3.39–3.34 (m, 1H, OCH_2_CH_2_), 3.22 (t, *J* = 7.0 Hz, 2H, CH_2_N_3_), 2.68 (d, *J* = 8.9 Hz, 1H, OH), 1.58–1.45 (m, 4H, octyl CH_2_), 1.33–1.26 (m, 8H, octyl CH_2_); ^13^C NMR (126 MHz, CDCl_3_, δ_C_) 166.0, 165.9, 165.8, 165.7, 165.2, 164.9, 133.3, 133.2, 133.1, 132.9, 130.1, 130.0, 129.84, 129.77, 129.7, 129.6, 129.5, 129.4, 129.2, 129.1, 129.0, 128.9, 128.7, 128.5, 128.4, 128.3, 128.21, 128.17, 128.1, 105.5, 105.0, 101.0, 100.5, 83.4, 83.1, 82.6, 82.0, 81.9, 81.81, 81.77, 81.7, 77.6, 77.5, 73.0, 72.9, 72.2, 72.0, 71.80, 71.76, 69.9, 69.6, 69.5, 67.4, 67.2, 66.3, 65.1, 63.3, 62.8, 62.7, 51.4, 29.3, 29.1, 28.8, 26.7, 26.0; HRMS (ESI) Calcd for (M + Na) C_170_H_149_O_49_N_3_Na: 3038.9157. Found 3038.9152.

**8-Azidooctyl 2,3,6-tri-*O*-benzoyl-5-*O*-levulinoyl-β-D-galactofuranosyl-(1→5)-[2,3,4,6-tetra-*O*-benzoyl-β-D-glucopyranosyl-(1→6)]-2,3-di-*O*-benzoyl-β-D-galactofuranosyl-(1→5)-2,3,6-tri-*O*-benzoyl-β-D-galactofuranosyl-(1→5)-[2,3,4,6-tetra-*O*-benzoyl-β-D-glucopyranosyl-(1→6)]-2,3-di-*O*-benzoyl-β-D-galactofuranosyl-(1→5)-2,3,6-tri-*O*-benzoyl-β-D-galactofuranosyl-(1→5)-[2,3,4,6-tetra-*O*-benzoyl-β-D-glucopyranosyl-(1→6)]-2,3-di-*O*-benzoyl-β-D-galactofuranoside (17)**. A mixture of alcohol **16** (69 mg, 23 μmol), thioglycoside **12** (57 mg, 35 μmol) and 4 Å molecular sieves (powder, 0.25 g) in CH_2_Cl_2_ (0.8 mL) was stirred at room temperature for 30 min and then cooled to –5 °C before silver trifluoromethanesulfonate (10 mg, 32 μmol) and *N*-iodosuccinimide (8 mg, 32 μmol) were added successively. After stirring at –5 °C for 4 h, triethylamine was added and the resulting mixture was filtered through Celite. The filtrate was diluted with CH_2_Cl_2_ (10 mL), washed with satd NaHCO_3_ aq soln and the organic layer was separated. The aqueous layer was extracted with CH_2_Cl_2_ (10 mL × 3) and the combined organic layers were dried over Na_2_SO_4_, filtered and concentrated. The residue was purified by chromatography (hexanes–acetone, 2:3) to afford 17 (88 mg, 85%) as a white foam. *R*_f_ 0.15 (hexanes–acetone, 2:3); [α]_D_ –11.3 (*c* 1.6, CHCl_3_); ^1^H NMR (500 MHz, CDCl_3_, δ_H_) δ 8.12–8.04 (m, 6H), 8.00–7.97 (m, 4H), 7.96–7.89 (m, 10H), 7.89–7.85 (m, 8H), 7.84–7.77 (m, 12H), 7.76–7.71 (m, 11H), 7.69–7.64 (m, 3H), 7.63–7.60 (m, 2H), 7.51–7.41 (m, 15H), 7.41–7.38 (m, 9H), 7.37–7.30 (m, 24H), 7.26–7.17 (m, 18H), 7.12–7.05 (m, 13H), 5.93 (app t, *J* = 9.5 Hz, 1H), 5.87–5.79 (m, 2H), 5.79–5.74 (m, 3H), 5.71 (s, 2H), 5.69–5.45 (m, 14H), 5.43 (d, *J* = 1.8 Hz, 1H), 5.39–5.33 (m, 2H), 5.09 (s, 1H), 5.05 (d, *J* = 7.8 Hz, 1H), 4.89 (d, *J* = 7.8 Hz, 1H), 4.82–4.81 (m, 2H), 4.72 (d, *J* = 4.9 Hz, 1H), 4.70–4.57 (m, 6H), 4.57–4.38 (m, 11H), 4.38–4.16 (m, 9H), 4.13–4.05 (m, 3H), 4.03–3.99 (m, 2H), 3.97–3.91 (m, 1H), 3.65–3.58 (m, 1H, OCH_2_CH_2_), 3.36–3.31 (m, 1H, OCH_2_CH_2_), 3.21 (t, *J* = 7.0 Hz, 2H, CH_2_N_3_), 2.57–2.46 (m, 2H, Lev CH_2_), 2.41–2.31 (m, 2H, Lev CH_2_), 1.95 (s, 3H, Lev CH_3_), 1.54–1.50 (m, 4H, octyl CH_2_), 1.28–1.27 (m, 8H, octyl CH_2_); ^13^C NMR (126 MHz, CDCl_3_, δ_C_) 206.0, 172.0, 171.9, 171.84, 171.78, 166.1, 166.0, 165.92, 165.86, 165.82, 165.79, 165.68, 165.67, 165.6, 165.54, 165.47, 165.4, 165.43, 165.42, 165.3, 165.2, 165.14, 165.09, 165.02, 164.98, 164.9, 164.8, 164.7, 133.3, 133.0, 132.9, 130.1, 130.03, 129.95, 129.8, 129.74, 129.66, 129.6, 129.5, 129.3, 129.0, 128.9, 128.7, 128.6, 128.5, 128.4, 128.3, 128.22, 128.17, 128.1, 105.8, 105.7, 105.5, 105.4, 105.0, 104.9, 101.13, 101.06, 100.5, 92.3, 83.2, 81.9, 81.8, 81.72, 81.69, 81.66, 81.6, 73.7, 73.5, 73.4, 73.1, 73.0, 72.9, 72.2, 72.14, 72.05, 71.9, 71.8, 71.7, 70.20, 70.18, 70.11, 70.07, 69.9, 69.7, 69.52, 69.46, 67.2, 63.6, 63.5, 63.3, 63.17, 63.15, 63.0, 62.92, 62.88, 62.8, 62.67, 62.66, 36.6, 29.7, 29.4, 29.3113, 29.3106, 29.3, 28.4, 26.7, 26.0, 24.7, 23.3; HRMS (ESI) Calcd for (M + Na) C_256_H_221_O_75_N_3_Na: 4559.3469. Found 4559.5341.

**8-Azidooctyl 2,3,6-tri-*O*-benzoyl-β-D-galactofuranosyl-(1→5)-[2,3,4,6-tetra-*O*-benzoyl-β-D-glucopyranosyl-(1→6)]-2,3-di-*O*-benzoyl-β-D-galactofuranosyl-(1→5)-2,3,6-tri-*O*-benzoyl-β-D-galactofuranosyl-(1→5)-[2,3,4,6-tetra-*O*-benzoyl-β-D-glucopyranosyl-(1→6)]-2,3-di-*O*-benzoyl-β-D-galactofuranosyl-(1→5)-2,3,6-tri-*O*-benzoyl-β-D-galactofuranosyl-(1→5)-[2,3,4,6-tetra-*O*-benzoyl-β-D-glucopyranosyl-(1→6)]-2,3-di-*O*-benzoyl-β-D-galactofuranoside (18)**. Nonasaccharide **17** (88 mg, 19 μmol) and hydrazine acetate (4 mg, 77 μmol) were dissolved in CH_2_Cl_2_–CH_3_OH (0.7:1.4 mL). After stirring at room temperature for 4 h, the mixture was diluted with CH_2_Cl_2_ (10 mL), and washed with a satd aq NaCl soln. The organic layer was separated and the aqueous layer was extracted with CH_2_Cl_2_ (10 mL × 3). The combined organic layers were dried over Na_2_SO_4_, filtered and concentrated. The residue was purified by chromatography (hexanes–EtOAc, 2:3) to afford 18 (80 mg, 78%) as a white foam. *R*_f_ 0.24 (hexanes–EtOAc, 1:1); [α]_D_ –14.3 (*c* 0.21, CHCl_3_); ^1^H NMR (600 MHz, CDCl_3_, δ_H_) 8.08–8.01 (m, 6H), 7.99–7.96 (m, 4H), 7.96–7.92 (m, 4H), 7.91–7.89 (m, 4H), 7.89–7.84 (m, 9H), 7.84–7.82 (m, 2H), 7.82–7.77 (m, 6H), 7.76–7.75 (m, 2H), 7.75–7.70 (m, 10H), 7.70–7.65 (m, 4H), 7.63–7.60 (m, 2H), 7.60–7.55 (m, 1H), 7.51–7.40 (m, 12H), 7.40–7.36 (m, 7H), 7.36–7.26 (m, 26H), 7.26–7.18 (m, 16H), 7.18–7.11 (m, 7H), 7.11–7.03 (m, 11H), 7.03–6.98 (m, 2H), 5.93 (app t, *J* = 9.5 Hz, 1H), 5.86–5.81 (m, 2H), 5.71–5.62 (m, 2H), 5.72–5.62 (m, 7H), 5.62–5.58 (m, 4H), 5.57–5.53 (m, 5H), 5.53–5.43 (m, 4H), 5.35 (d, *J* = 1.5 Hz, 1H), 5.08 (s, 1H), 5.05 (d, *J* = 7.8 Hz, 1H), 4.88 (d, *J* = 7.9 Hz, 1H), 4.85 (d, *J* = 7.8 Hz, 1H), 4.81 (dd, *J* = 4.9, 2.7 Hz, 1H), 4.70 (dd, *J* = 4.8, 2.4 Hz, 1H), 4.67 (dd, *J* = 11.8, 3.6 Hz, 1H), 4.62–4.59 (m, 3H), 4.56–4.38 (m, 13H), 4.34–4.23 (m, 5H), 4.22–4.18 (m, 2H), 4.10–4.05 (m, 4H), 4.01–3.95 (m, 3H), 3.61 (dt, *J* = 9.6, 6.6 Hz, 1H, OCH_2_CH_2_), 3.35 (dt, *J* = 9.6, 5.9 Hz, 1H, OCH_2_CH_2_), 3.20 (t, *J* = 7.0 Hz, 2H, CH_2_N_3_), 1.58–1.46 (m, 5H, OH, octyl CH_2_), 1.31–1.27 (m, 8H, octyl CH_2_); ^13^C NMR (151 MHz, CDCl_3_, δ_C_) 166.0, 165.9, 165.69, 165.66, 165.51, 165.45, 165.23, 165.18, 165.1, 165.0, 164.7, 133.4, 133.32, 133.27, 133.1, 132.9, 132.8, 130.11, 130.05, 130.0, 129.88, 129.85, 129.8, 129.74, 129.71, 129.69, 129.64, 129.58, 129.55, 129.53, 129.45, 129.3, 129.14, 129.10, 129.03, 129.00, 128.95, 128.91, 128.88, 128.73, 128.71, 128.69, 128.59, 128.55, 128.5, 128.44, 128.38, 128.36, 128.33, 128.29, 128.25, 128.23, 128.19, 128.15, 128.1, 105.7, 105.6, 105.5, 105.1, 105.0, 101.1, 101.0, 100.5, 83.3, 83.2, 82.3, 82.2, 81.9, 81.7, 81.6, 77.7, 73.9, 73.8, 73.6, 73.5, 73.1, 73.00, 72.96, 72.2, 72.1, 72.0, 71.8, 70.8, 69.9, 69.6, 67.2, 66.3, 65.8, 65.1, 63.3, 62.9, 62.7, 51.4, 29.7, 29.4, 29.1, 28.8, 26.7, 26.1; HRMS (ESI) Calcd for (M + Na) C_251_H_215_O_73_N_3_Na: 4464.4453. Found: 4463.4397.

**8-Azidooctyl 2,3,6-tri-*O*-benzoyl-5-*O*-levulinoyl-β-D-galactofuranosyl-(1→5)-[2,3,4,6-tetra-*O*-benzoyl-β-D-glucopyranosyl-(1→6)]-2,3-di-*O*-benzoyl-β-D-galactofuranosyl-(1→5)-2,3,6-tri-*O*-benzoyl-β-D-galactofuranosyl-(1→5)-[2,3,4,6-tetra-*O*-benzoyl-β-D-glucopyranosyl-(1→6)]-2,3-di-*O*-benzoyl-β-D-galactofuranosyl-(1→5)-2,3,6-tri-*O*-benzoyl-β-D-galactofuranosyl-(1→5)-[2,3,4,6-tetra-*O*-benzoyl-β-D-glucopyranosyl-(1→6)]-2,3-di-*O*-benzoyl-β-D-galactofuranosyl-(1→5)-2,3,6-tri-*O*-benzoyl-β-D-galactofuranosyl-(1→5)-[2,3,4,6-tetra-*O*-benzoyl-β-D-glucopyranosyl-(1→6)]-2,3-di-*O*-benzoyl-β-D-galactofuranoside (19)**. A mixture of alcohol **18** (71 mg, 16 μmol), glycosyl fluoride **24** (86 mg, 56 μmol) and 4 Å molecular sieves (powder, 0.25 g) in CH_2_Cl_2_ (0.8 mL) was stirred at room temperature for 30 min and then cooled to 0 °C before bis(cyclopentadienyl)zirconium (IV) dichloride (16 mg, 55 μmol) and silver trifluoromethanesulfonate (28 mg, 11 μmol) were added successively. After stirring for 4 h at rt, triethylamine was added and the resulting mixture was filtered through Celite. The filtrate was diluted with CH_2_Cl_2_ (10 mL) and washed with satd NaHCO_3_ soln. The organic layer was separated and the aqueous layer was extracted with CH_2_Cl_2_ (10 mL × 3). The combined organic layer was dried over Na_2_SO_4_, filtered and the filtrate was concentrated. The residue was purified by chromatography (hexanes–acetone, 2:3) to afford 19 (81 mg, 85%) as a white foam. *R*_f_ 0.1 (hexanes–acetone, 2:3); [α]_D_ –18.8 (*c* 0.48, CHCl_3_); ^1^H NMR (600 MHz, CDCl_3_, δ_H_) 8.11–8.02 (m, 8H), 8.02–7.68 (m, 56H), 7.68–7.63 (m, 4H), 7.63–7.58 (m, 4H), 7.58–7.53 (m, 1H), 7.50–7.27 (m, 52H), 7.25–7.15 (m, 31H), 7.15–6.96 (m, 24H), 5.96–5.92 (m, 1H), 5.86–5.78 (m, 3H), 5.78–5.57 (m, 18H), 5.57–5.45 (m, 11H), 5.45–5.41 (m, 1H), 5.39–5.34 (m, 2H), 5.09 (s, 1H), 5.06 (d, *J* = 8.6 Hz, 1H), 4.91 (d, *J* = 7.8 Hz, 1H), 4.85 (d, *J* = 7.7 Hz, 1H), 4.83–4.81 (m, 2H), 4.71–4.66 (m, 3H), 4.65–4.17 (m, 29H), 4.12–4.07 (m, 5H), 4.06–3.98 (m, 3H), 4.04–3.99 (m, 2H), 3.64–3.58 (m, 1H, OCH_2_CH_2_), 3.35 (d, *J* = 8.5 Hz, 1H, OCH_2_CH_2_), 3.20 (t, *J* = 7.0 Hz, 2H, CH_2_N_3_), 2.58–2.22 (m, 4H, Lev CH_2_), 1.94 (s, 3H, Lev CH_3_), 1.55–1.48 (m, 4H, octyl CH_2_), 1.33–1.28 (m, 8H, octyl CH_2_); ^13^C NMR (151 MHz, CDCl_3_, δ_C_) δ 206.0, 171.2, 165.91, 165.87, 165.68, 165.66, 165.50, 165.45, 165.3, 165.23, 165.16, 165.1, 165.02, 164.97, 164.8, 164.7, 133.4, 133.3, 133.1, 132.9, 132.7, 130.14, 130.07, 130.0, 129.9, 129.8, 129.7, 129.64, 129.58, 129.53, 129.46, 129.2, 129.14, 129.10, 129.02, 128.99, 128.93, 128.91, 128.88, 128.8, 128.7, 128.63, 128.55, 128.5, 128.4, 128.34, 128.30, 128.26, 128.2, 128.14, 128.09, 105.9, 105.8, 105.7, 105.5, 105.1, 105.0, 101.2, 101.1, 100.5, 83.3, 82.8, 82.6, 82.2, 82.0, 81.9, 81.7, 81.5, 77.9, 77.7, 76.5, 74.3, 73.7, 73.6, 73.1, 73.0, 72.2, 72.1, 71.8, 70.2, 69.9, 69.8, 69.7, 69.5, 67.2, 65.9, 65.1, 63.7, 63.3, 62.8, 62.7, 51.4, 29.7, 29.6, 29.34, 29.29, 29.1, 28.8, 27.8, 26.1; HRMS (ESI) Calcd for (M + Na) C_337_H_287_O_99_N_3_Na:5985.9507. Found 5985.9546.

**8-Azidooctyl 2,3,6-tri-*O*-benzoyl-β-D-galactofuranosyl-(1→5)-[2,3,4,6-tetra-*O*-benzoyl-β-D-glucopyranosyl-(1→6)]-2,3-di-*O*-benzoyl-β-D-galactofuranosyl-(1→5)-2,3,6-tri-*O*-benzoyl-β-D-galactofuranosyl-(1→5)-[2,3,4,6-tetra-*O*-benzoyl-β-D-glucopyranosyl-(1→6)]-2,3-di-*O*-benzoyl-β-D-galactofuranosyl-(1→5)-2,3,6-tri-*O*-benzoyl-β-D-galactofuranosyl-(1→5)-[2,3,4,6-tetra-*O*-benzoyl-β-D-glucopyranosyl-(1→6)]-2,3-di-*O*-benzoyl-β-D-galactofuranosyl-(1→5)-2,3,6-tri-*O*-benzoyl-β-D-galactofuranosyl-(1→5)-[2,3,4,6-tetra-*O*-benzoyl-β-D-glucopyranosyl-(1→6)]-2,3-di-*O*-benzoyl-β-D-galactofuranoside (20)**. Dodecasaccharide **19** (60 mg, 10 μmol) and hydrazine acetate (1 mg, 11 μmol) were dissolved in CH_2_Cl_2_–CH_3_OH (0.9:0.3 mL). After stirring at room temperature for 4 h, the resulting mixture was diluted with CH_2_Cl_2_ (10 mL) and washed with satd NaCl solution. The organic layer was separated and the aqueous layer was extracted with CH_2_Cl_2_ (10 mL × 3). The combined organic layer was dried over Na_2_SO_4_, filtered, and the filtrate was concentrated. The residue was purified by chromatography (hexanes–EtOAc, 2:3) to afford 20 (44 mg, 70%) as a white foam. *R*_f_ 0.15 (hexanes–EtOAc, 1:1); [α]_D_ –18.6 (*c* 0.43, CHCl_3_); ^1^H NMR (600 MHz, CDCl_3_, δ_H_) 8.10–8.04 (m, 7H), 8.03–7.95 (m, 9H), 7.94–7.80 (m, 26H), 7.78–7.68 (m, 26H), 7.66–7.58 (m, 5H), 7.52–7.29 (m, 55H), 7.28–7.17 (m, 29H), 7.15–7.00 (m, 23H), 5.98–5.94 (m, 1H), 5.89–5.81 (m, 4H), 5.80–5.66 (m, 12H), 5.66–5.61 (m, 7H), 5.59–5.45 (m, 12H), 5.38 (d, *J* = 4.2 Hz, 1H), 5.11 (d, *J* = 4.4 Hz, 1H), 5.08 (app t, *J* = 6.4 Hz, 1H), 4.93 (app t, *J* = 6.4 Hz, 1H), 4.89–4.83 (m, 3H), 4.75–4.62 (m, 5H), 4.61–4.38 (m, 19H), 4.36–4.20 (m, 9H), 4.15–4.11 (m, 5H), 4.05–3.96 (m, 4H), 3.65–3.63 (m, 1H, OCH_2_CH_2_), 3.41–3.34 (m, 1H, OCH_2_CH_2_), 3.22 (t, *J* = 7.0 Hz, 2H, CH_2_N_3_), 1.59–1.50 (m, 4H, octyl CH_2_), 1.36–1.30 (m, 8H, octyl CH_2_); ^13^C NMR (151 MHz, CDCl_3_, δ_C_) 166.2, 166.0, 165.84, 165.82, 165.63, 165.61, 165.49, 165.47, 165.5, 165.44, 165.41, 165.3, 165.2, 165.14, 165.11, 165.0, 164.92, 164.88, 164.69, 164.66, 133.5, 133.32, 133.28, 133.2, 133.1, 133.0, 132.9, 132.8, 132.7, 132.6, 130.1, 130.0, 129.9, 129.81, 129.77, 129.7, 129.64, 129.59, 129.53, 129.48, 129.2, 129.1, 129.04, 129.01, 128.97, 128.93, 128.89, 128.88, 128.81, 128.77, 128.7, 128.6, 128.52, 128.50, 128.45, 128.4, 128.32, 128.29, 128.25, 128.21, 128.15, 128.0, 105.7, 105.60, 105.55, 105.5, 105.4, 105.0, 104.9, 101.1, 101.01, 101.00, 100.4, 83.2, 83.1, 82.7, 82.5, 82.2, 82.1, 81.94, 81.86, 81.8, 81.7, 81.6, 81.5, 81.4, 77.8, 77.72, 77.65, 76.7, 76.63, 76.61, 76.5, 76.4, 74.1, 73.92, 73.86, 73.7, 73.5, 73.4, 73.13, 73.07, 73.0, 72.9, 72.1, 72.0, 71.9, 71.80, 71.75, 71.6, 71.23, 71.22, 71.19, 70.6, 69.9, 69.7, 69.6, 69.5, 67.1, 66.2, 65.9, 65.1, 63.2, 62.9, 62.8, 62.6, 51.4, 29.7, 29.3, 29.1, 28.8, 26.7, 26.0; HRMS (ESI) Calcd for (M + Na) C_332_H_281_O_97_N_3_Na: 5887.8491. Found 5886.8447.

**8-Azidooctyl 2,3,6-tri-*O*-benzoyl-5-*O*-levulinoyl-β-D-galactofuranosyl-(1→5)-[2,3,4,6-tetra-*O*-benzoyl-β-D-glucopyranosyl-(1→6)]-2,3-di-*O*-benzoyl-β-D-galactofuranosyl-(1→5)-2,3,6-tri-*O*-benzoyl-β-D-galactofuranosyl-(1→5)-[2,3,4,6-tetra-*O*-benzoyl-β-D-glucopyranosyl-(1→6)]-2,3-di-*O*-benzoyl-β-D-galactofuranosyl-(1→5)-2,3,6-tri-*O*-benzoyl-β-D-galactofuranosyl-(1→5)-[2,3,4,6-tetra-*O*-benzoyl-β-D-glucopyranosyl-(1→6)]-2,3-di-*O*-benzoyl-β-D-galactofuranosyl-(1→5)-2,3,6-tri-*O*-benzoyl-β-D-galactofuranosyl-(1→5)-[2,3,4,6-tetra-*O*-benzoyl-β-D-glucopyranosyl-(1→6)]-2,3-di-*O*-benzoyl-β-D-galactofuranosyl-(1→5)-2,3,6-tri-*O*-benzoyl-β-D-galactofuranosyl-(1→5)-[2,3,4,6-tetra-*O*-benzoyl-β-D-glucopyranosyl-(1→6)]-2,3-di-*O*-benzoyl-β-D-galactofuranoside (21)**. A mixture of alcohol **20** (40 mg, 6.8 μmol), glycosyl fluoride **24** (53 mg, 34 μmol) and 4 Å molecular sieves (powder, 0.40 g) in CH_2_Cl_2_ (1.2 mL) was stirred at room temperature for 30 min and then cooled to 0 °C before bis(cyclopentadienyl)zirconium (IV) dichloride (10 mg, 34 μmol) and silver trifluoromethanesulfonate (18 mg, 68 μmol) were added successively. After stirring for 4h at rt, triethylamine was added and the resulting mixture was filtered through Celite. The filtrate was diluted with CH_2_Cl_2_ (10 mL) and washed with satd NaHCO_3_ soln. The organic layer was separated and the aqueous layer was extracted with CH_2_Cl_2_ (10 mL × 3). The combined organic layers were dried over Na_2_SO_4_, filtered, the filtrate was concentrated and the residue was purified by chromatography (hexanes–acetone, 1:1.17). The resulting compounds was purified again by LH-20 size exclusion chromatography (180 mL, CH_2_Cl_2_–CH_3_OH, 1:3) to afford 21 (43 mg, 87%) as a white foam. *R*_f_ 0.3 (hexanes–acetone, 1:1.17); [α]_D_ –12.9 (*c* 1.63, CHCl_3_); ^1^H NMR (600 MHz, CDCl_3_, δ_H_) δ 8.15–7.99 (m, 10H), 7.97–7.95 (m, 4H), 7.94–7.52 (m, 78H), 7.51–7.27 (m, 61H), 7.25–6.92 (m, 72H), 5.91 (app t, *J* = 9.5 Hz, 1H), 5.84–5.76 (m, 4H), 5.76–5.71 (m, 2H), 5.71–5.65 (m, 8H), 5.64–5.56 (m, 13H), 5.55–5.43 (m, 15H), 5.40 (d, *J* = 1.9 Hz, 1H), 5.34–5.33 (m, 2H), 5.06 (s, 1H), 5.03 (d, *J* = 7.8 Hz, 1H), 4.88 (d, *J* = 7.8 Hz, 1H), 4.86–4.76 (m, 4H), 4.73–4.57 (m, 6H), 4.57–4.46 (m, 10H), 4.44–4.34 (m, 15H), 4.33–4.26 (m, 6H), 4.25–4.16 (m, 4H), 4.11–4.04 (m, 4H), 4.04–3.96 (m, 4H), 3.95–3.92 (m, 3H), 3.59 (dt, *J* = 9.6, 6.5 Hz, 1H, OCH_2_CH_2_), 3.33 (dt, *J* = 9.5, 6.3 Hz, 1H, OCH_2_CH_2_), 3.19 (t, *J* = 7.0 Hz, 2H, CH_2_N_3_), 2.53–2.11 (m, 1H, Lev CH_2_), 2.39 – 2.24 (m, 2H, Lev CH_2_), 2.20 – 2.03 (m, 1H, Lev CH_2_), 1.93 (s, 3H, Lev CH_3_), 1.56–1.37 (m, 4H, octyl CH_2_), 1.31–1.23 (m, 8H, octyl CH_2_); ^13^C NMR (151 MHz, CDCl_3_, δ_C_) δ 206.0, 165.9, 165.8, 165.6, 165.50, 165.45, 165.4, 165.3, 165.1, 165.0, 164.7, 133.3, 133.2, 133.0, 132.8, 132.6, 130.1, 130.0, 129.9, 129.8, 129.74, 129.71, 129.66, 129.6, 129.54, 129.49, 129.4, 129.3, 129.2, 129.04, 128.97, 128.9, 128.71, 128.66, 128.6, 128.5, 128.4, 128.32, 128.29, 128.23, 128.16, 128.1, 128.0, 105.83, 105.79, 105.7, 105.60, 105.59, 105.5, 105.4, 105.3, 105.0, 104.9, 101.1, 101.0, 100.4, 83.20, 83.15, 82.78, 82.75, 82.5, 82.4, 82.2, 82.0, 81.9, 81.8, 81.71, 81.67, 81.6, 81.5, 77.9, 77.82, 77.75, 77.5, 76.6, 76.42, 76.40, 76.3, 76.2, 74.3, 74.2, 74.0, 73.7, 73.58, 73.56, 73.5, 73.4, 73.2, 73.11, 73.08, 72.9, 72.1, 72.03, 72.02, 71.80, 71.76, 70.1, 69.9, 69.7, 69.63, 69.58, 69.4, 67.1, 65.92, 65.86, 65.1, 63.6, 63.2, 62.9, 62.8, 62.7, 62.6, 51.4, 37.9, 29.6, 29.3, 29.1, 28.8, 27.8, 26.7, 26.0; HRMS (ESI) Calcd for (M + Na) C_418_H_353_O_123_N_3_Na: 7409.3550. Found 7409.3800.

**8-Azidooctyl 2,3,6-tri-*O*-benzoyl-β-D-galactofuranosyl-(1→5)-[2,3,4,6-tetra-*O*-benzoyl-β-D-glucopyranosyl-(1→6)]-2,3-di-*O*-benzoyl-β-D-galactofuranosyl-(1→5)-2,3,6-tri-*O*-benzoyl-β-D-galactofuranosyl-(1→5)-[2,3,4,6-tetra-*O*-benzoyl-β-D-glucopyranosyl-(1→6)]-2,3-di-*O*-benzoyl-β-D-galactofuranosyl-(1→5)-2,3,6-tri-*O*-benzoyl-β-D-galactofuranosyl-(1→5)-[2,3,4,6-tetra-*O*-benzoyl-β-D-glucopyranosyl-(1→6)]-2,3-di-*O*-benzoyl-β-D-galactofuranosyl-(1→5)-2,3,6-tri-*O*-benzoyl-β-D-galactofuranosyl-(1→5)-[2,3,4,6-tetra-*O*-benzoyl-β-D-glucopyranosyl-(1→6)]-2,3-di-*O*-benzoyl-β-D-galactofuranosyl-(1→5)-2,3,6-tri-*O*-benzoyl-β-D-galactofuranosyl-(1→5)-[2,3,4,6-tetra-*O*-benzoyl-β-D-glucopyranosyl-(1→6)]-2,3-di-*O*-benzoyl-β-D-galactofuranoside (22)**. Pentadecasaccharide **21** (20 mg, 2.8 μmol) and hydrazine acetate (0.3 mg, 3.4 μmol) were dissolved in CH_2_Cl_2_–CH_3_OH (0.3:0.1 mL). After stirring at room temperature for 4 h, the resulting mixture was diluted with CH_2_Cl_2_ (10 mL) and washed with satd NaCl soln. The organic layer was separated and the aqueous layer was extracted with CH_2_Cl_2_ (10 mL × 3). The combined organic layer was dried over Na_2_SO_4_, filtered, the filtrate was concentrated. The residue was purified by chromatography (hexanes–acetone, 1:1.17) to give a compound that was purified again by LH-20 size exclusion chromatography (180 mL, CH_2_Cl_2_–CH_3_OH, 1:3) affording 22 (16 mg, 87%) as a white foam. *R*_f_ 0.38 (hexanes–acetone, 1:1.17); [α]_D_ –8.2 (*c* 1.1, CHCl_3_); ^1^H NMR (600 MHz, CDCl_3_, δ_H_) 8.05–7.99 (m, 10H), 7.98–7.90 (m, 9H), 7.90–7.82 (m, 21H), 7.81–7.76 (m, 12H), 7.75–7.62 (m, 34H), 7.61–7.53 (m, 7H), 7.49–7.27 (m, 60H), 7.24–6.94 (m, 72H), 5.91 (app t, *J* = 9.5 Hz, 1H), 5.85–5.71 (m, 6H), 5.71–5.55 (m, 20H), 5.55–5.40 (m, 16H), 5.33 (d, *J* = 1.6 Hz, 1H), 5.06 (s, 1H), 5.03 (d, *J* = 7.7 Hz, 1H), 4.88 (d, *J* = 7.8 Hz, 1H), 4.85–4.77 (m, 4H), 4.71–4.56 (m, 6H), 4.56–4.33 (m, 23H), 4.33–4.15 (m, 12H), 4.12–3.90 (m, 13H), 3.59 (dt, *J* = 9.6, 6.6 Hz, 1H, OCH_2_CH_2_), 3.33 (dt, *J* = 9.7, 6.3 Hz, 1H, OCH_2_CH_2_), 3.18 (t, *J* = 7.0 Hz, 2H, CH_2_N_3_), 2.72 (d, *J* = 8.5 Hz, 1H, OH), 1.55–1.45 (m, 4H, octyl CH_2_), 1.36–1.28 (m, 8H, octyl CH_2_); ^13^C NMR (151 MHz, CDCl_3_, δ_C_) δ 166.2, 166.0, 165.9, 165.7, 165.51, 165.45, 165.4, 165.3, 165.23, 165.18, 165.15, 165.0, 164.94, 164.90, 164.71, 164.67, 133.32, 133.26, 133.2, 133.0, 132.9, 132.8, 132.7, 132.6, 130.1, 130.04, 130.98, 130.95, 129.8, 129.7, 129.6, 129.5, 129.1, 129.03, 128.97, 128.9, 128.8, 128.7, 128.6, 128.52, 128.47, 128.42, 128.35, 128.3, 128.22, 128.16, 128.10, 128.06, 105.81, 105.77, 105.75, 105.62, 105.60, 105.5, 105.44, 105.41, 105.1, 104.9, 101.2, 101.0, 100.5, 83.24, 83.15, 83.1, 82.8, 82.54, 82.45, 82.23, 82.21, 82.20, 82.01, 81.96, 81.91, 81.86, 81.69, 81.65, 81.5, 77.73, 77.68, 76.5, 74.2, 74.1, 74.0, 73.73, 73.70, 73.62, 73.57, 73.49, 73.45, 73.2, 73.12, 73.07, 73.0, 72.2, 72.1, 71.94, 71.85, 71.8, 71.7, 71.38, 71.35, 71.3, 71.2, 69.9, 69.8, 69.69, 69.65, 69.6, 67.2, 66.3, 65.92, 65.86, 65.1, 63.4, 63.3, 62.9, 62.8, 62.7, 62.8, 51.4, 29.7, 29.3, 29.1, 28.8, 26.7, 26.0; HRMS (ESI) Calcd for (M + Na) C_413_H_347_O_121_N_3_Na:7311.2529. Found 7311.2597.

**8-Azidooctyl 2,3,6-tri-*O*-benzoyl-5-*O*-levulinoyl-β-D-galactofuranosyl-(1→5)-[2,3,4,6-tetra-*O*-benzoyl-β-D-glucopyranosyl-(1→6)]-2,3-di-*O*-benzoyl-β-D-galactofuranosyl-(1→5)-2,3,6-tri-*O*-benzoyl-β-D-galactofuranosyl-(1→5)-[2,3,4,6-tetra-*O*-benzoyl-β-D-glucopyranosyl-(1→6)]-2,3-di-*O*-benzoyl-β-D-galactofuranosyl-(1→5)-2,3,6-tri-*O*-benzoyl-β-D-galactofuranosyl-(1→5)-[2,3,4,6-tetra-*O*-benzoyl-β-D-glucopyranosyl-(1→6)]-2,3-di-*O*-benzoyl-β-D-galactofuranosyl-(1→5)-2,3,6-tri-*O*-benzoyl-β-D-galactofuranosyl-(1→5)-[2,3,4,6-tetra-*O*-benzoyl-β-D-glucopyranosyl-(1→6)]-2,3-di-*O*-benzoyl-β-D-galactofuranosyl-(1→5)-2,3,6-tri-*O*-benzoyl-β-D-galactofuranosyl-(1→5)-[2,3,4,6-tetra-*O*-benzoyl-β-D-glucopyranosyl-(1→6)]-2,3-di-*O*-benzoyl-β-D-galactofuranosyl-(1→5)-2,3,6-tri-*O*-benzoyl-β-D-galactofuranosyl-(1→5)-[2,3,4,6-tetra-*O*-benzoyl-β-D-glucopyranosyl-(1→6)]-2,3-di-*O*-benzoyl-β-D-galactofuranoside (23)**. A mixture of alcohol **22** (16 mg, 2.2 μmol), glycosyl fluoride **24** (16 mg, 11 μmol) and 4 Å molecular sieves (powder, 52 mg) in CH_2_Cl_2_ (0.2 mL) was stirred at room temperature for 30 min and then cooled to 0 °C before bis(cyclopentadienyl)zirconium (IV) dichloride (3.2 mg, 11 μmol) and silver trifluoromethanesulfonate (5.6 mg, 22 μmol) were added successively. After stirring for 4h at rt, triethylamine was added and the resulting mixture was filtered through Celite. The filtrate was diluted with CH_2_Cl_2_ (10 mL) and washed with a satd NaHCO_3_ soln. The organic layer was separated and the aqueous layer was extracted with CH_2_Cl_2_ (10 mL × 3). The combined organic layers were dried over Na_2_SO_4_, filtered and the filtrate was concentrated. The residue was purified by chromatography (hexanes–acetone, 1:1.17) to give a compound that was purified again by LH-20 size exclusion chromatography (180 mL, CH_2_Cl_2_–CH_3_OH, 1:3) to afford 23 (14 mg, 74%) as a white foam. *R*_f_ 0.13 (hexanes–acetone, 1:1.17); [α]_D_ –37.5 (*c* 0.08, CHCl_3_); ^1^H NMR (500 MHz, CDCl_3_, δ_H_) 8.18–7.58 (m, 109H), 7.58–6.95 (m, 161H), 5.94 (app t, *J* = 9.6 Hz, 1H), 5.90–5.46 (m, 51H), 5.43 (s, 1H), 5.37 (d, *J* = 5.7 Hz, 2H), 5.09 (s, 1H), 5.06 (d, *J* = 7.8 Hz, 1H), 4.91 (d, *J* = 7.8 Hz, 1H), 4.90–4.79 (m, 4H), 4.76–4.20 (m, 49H), 4.13–3.90 (m, 15H), 3.72–3.70 (m, 2H, OCH_2_CH_2_), 3.51 (t, *J* = 6.8 Hz, 2H, CH_2_N_3_), 2.60–2.24 (m, 4H, Lev CH_2_), 1.95 (s, 3H, Lev CH_3_), 1.68–1.52 (m, 4H, octyl CH_2_), 1.54–1.35 (m, 8H, octyl CH_2_); ^13^C NMR (126 MHz, CDCl_3_, δ_C_) 206.0, 171.8, 171.2, 166.0, 165.9, 165.6, 165.4, 165.3, 165.2, 165.0, 164.7, 133.2, 133.0, 132.9, 132.6, 130.1, 129.8, 129.7, 129.6, 129.5, 129.1, 128.9, 128.7, 128.7, 128.6, 128.3, 128.2, 128.1, 105.8, 105.7, 105.6, 105.5, 105.0, 101.1, 101.0, 100.5, 99.8, 83.2, 81.8, 81.7, 81.5, 73.2, 73.1, 72.9, 72.5, 72.1, 71.8, 71.8, 71.3, 70.5, 70.2, 69.9, 69.7, 69.6, 69.6, 69.5, 69.5, 67.2, 65.9, 61.9, 60.4, 51.4, 37.9, 29.7, 29.4, 29.3, 29.3; HRMS (ESI) Calcd for (M + Na) C_499_H_419_O_147_N_3_Na: 8832.7598. Found 8832.7477.

**2,3,6-Tri-*O*-benzoyl-5-*O*-levulinoyl-β-D-galactofuranosyl-(1→5)-[2,3,4,6-tetra-*O*-benzoyl-β-D-glucopyranosyl-(1→6)]-2,3-di-*O*-benzoyl-β-D-galactofuranosyl fluoride (24)**. To a solution of 12 (0.11 g, 65 μmol) in CH_2_Cl_2_ (1.2 mL) at 0 °C was added *N*,*N*-diethylamino sulfur trifluoride (24 μL, 182 μmol) followed by *N*-bromosuccinimide (33 mg, 185 μmol). The reaction mixture was stirred overnight at room temperature and then CH_3_OH was added. The reaction mixture was diluted with CH_2_Cl_2_ and washed with satd aq NaHCO_3_ soln before the organic layer was separated. The aqueous layer was extracted again with CH_2_Cl_2_ and the combined organic layers were dried over Na_2_SO_4_. The solution was filtered, the filtrate was concentrated and the resulting residue was purified by chromatography (hexanes–acetone, 5:2) to afford glycosyl fluoride 24 (0.1 g, 99%) as a white foam. *R*_f_ 0.13 (hexanes–acetone, 5:2); [α]_D_ +4.4 (*c* 3.2, CHCl_3_); ^1^H NMR (500 MHz, CDCl_3_, δ_H_) 8.13–8.08 (m, 2H, Ar), 7.99–7.91 (m, 8H, Ar), 7.91–7.87 (m, 2H, Ar), 7.82–7.74 (m, 6H, Ar), 7.62–7.53 (m, 2H, Ar), 7.52–7.42 (m, 7H, Ar), 7.42–7.38 (m, 4H, Ar), 7.37–7.29 (m, 10H, Ar), 7.27–7.18 (m, 2H, Ar), 7.15–7.09 (m, 2H, Ar), 5.92 (app t, *J* = 9.6 Hz, 1H, H-3’’), 5.88–5.71 (m, 2H, H-1, H-2), 5.67–5.59 (m, 2H, H-4’’, H-5’), 5.59–5.49 (m, 4H, H-1’, H-2’, H-3, H-2’’), 5.46–5.42 (m, 1H, H-3’), 5.04 (d, *J* = 7.8 Hz, 1H, H-1’’), 4.76 (app t, *J* = 4.8 Hz, 1H, H-4’), 4.69–4.58 (m, 2H, H-4, H-6b’), 4.56–4.78 (m, 2H, H-6b’’, H-5), 4.44–4.33 (m, 2H, H-6a’’, H-6a’), 4.24 (dd, *J* = 11.0, 5.2 Hz, 1H, H-6b), 4.18 (ddd, *J* = 9.9, 5.4, 3.2 Hz, 1H, H-5’’), 4.14–4.05 (m, 1H, H-6a), 2.66–2.36 (m, 4H, Lev CH_2_), 2.01 (s, 3H, Lev CH_3_); ^13^C NMR (126 MHz, CDCl_3_, δ_C_) 206.0 (Lev ketone C=O), 171.9 (Lev ester C=O ), 166.0 (Ar), 165.9 (Ar), 165.7 (Ar), 165.43 (Ar), 165.37 (Ar), 165.3 (Ar), 165.14 (Ar), 165.08 (Ar), 164.9 (Ar), 133.7 (Ar), 133.60 (Ar), 133.55 (Ar), 133.4 (Ar), 133.3 (Ar), 133.2 (Ar), 133.1 (Ar), 130.1 (Ar), 129.9 (Ar), 129.8 (Ar), 129.74 (Ar), 129.71 (Ar), 129.66 (Ar), 129.6 (Ar), 129.5 (Ar), 129.1 (Ar), 129.0 (Ar), 128.79 (Ar), 128.76 (Ar), 128.7 (Ar), 128.61 (Ar), 128.55 (Ar), 128.5 (Ar), 128.4 (Ar), 128.3 (Ar), 128.2 (Ar), 128.24 (Ar), 128.21 (Ar), 112.06 (d, *J* = 225.8 Hz, C-1), 104.8 (C-1’), 100.7 (C-1’’), 85.0 (C-4), 81.8 (C-4’), 81.4 (C-2’), 80.9 (d, *J* = 39.3 Hz, C-2), 77.1 (C-5), 75.7 (C-3’’), 73.2 (C-2’’), 72.8 (C-5’’), 72.3 (C-5’), 71.7 (C-4’’), 70.1 (C-6), 69.7 (C-3), 67.7(C-3’), 63.2 (C-6’), 63.11 (C-6’’), 37.9 (Lev CH_2_), 29.6 (Lev CH_2_), 27.9 (Lev CH_3_); ^19^F NMR (470 MHz, CDCl_3_, δ_F_) –124.28; HRMS (ESI) Calcd for (M + Na) C_86_H_73_FNaO_26_: 1563.4266. Found 1563.4272.
